# Huang-Lian-Jie-Du Decoction Ameliorates Acute Ulcerative Colitis in Mice *via* Regulating NF-κB and Nrf2 Signaling Pathways and Enhancing Intestinal Barrier Function

**DOI:** 10.3389/fphar.2019.01354

**Published:** 2019-11-21

**Authors:** Ziwen Yuan, Lihong Yang, Xiaosong Zhang, Peng Ji, Yongli Hua, Yanming Wei

**Affiliations:** Institute of Traditional Chinese Veterinary Medicine, College of Veterinary Medicine, Gansu Agricultural University, Lanzhou, China

**Keywords:** Huang-lian-Jie-du Decoction, ulcerative colitis, inflammation, oxidative stress, NF-κB, Nrf2, mucosal protection

## Abstract

Evidence shows that intestinal inflammation, oxidative stress, and injury of mucosal barrier are closely related to the pathogenesis of ulcerative colitis (UC). Huang-lian-Jie-du Decoction (HLJDD) is a well-known prescription of traditional Chinese medicine with anti-inflammatory and antioxidative activities, which may be used to treat UC. However, its therapeutic effect and mechanism are still unclear. In this study, the UC model of BABL/c mice were established by DSS [3.5% (w/v)], and HLJDD was given orally for treatment at the same time. During the experiment, the clinical symptoms of mice were scored by disease activity index (DAI). Besides, the effects of HLJDD on immune function, oxidative stress, colon NF-κB and Nrf2 signaling pathway, and intestinal mucosal barrier function in UC mice were also investigated. The results showed that HLJDD could alleviate body weight loss and DAI score of UC mice, inhibit colonic shortening and relieve colonic pathological damage, and reduce plasma and colon MPO levels. In addition, HLJDD treatment significantly up-regulated plasma IL-10, down-regulated TNF-α and IL-1β levels, and inhibited the expression of NF-κB p65, p-IκKα/β, and p-IκBα proteins in the colon. Moreover, NO and MDA levels in colon tissues were significantly reduced after HLJDD treatment, while GSH, SOD levels and Nrf2, Keap1 protein expression levels were remarkably elevated. Additionally, HLJDD also protected intestinal mucosa by increasing the secretion of mucin and the expression of ZO-1 and occludin in colonic mucosa. These results indicate that HLJDD could effectively alleviate DSS-induced mice UC by suppressing NF-κB signaling pathway, activating Nrf2 signaling pathway, and enhancing intestinal barrier function.

## Introduction

Ulcerative colitis (UC), a type of inflammatory bowel disease, mainly invades the rectum and colon (Ungaro et al., 2017). The pathological features of UC are inflammatory and ulcerative lesions of the mucosa and submucosa (Eom et al., 2018). Up to now, the specific pathogenesis of UC is still unclear. It is believed that the pathogenesis of UC is mainly related to individual genetic susceptibility, immune dysfunction, impaired intestinal epithelial cell integrity, and alteration of intestinal flora (Sonnenberg and Siegmund, 2016). Frequent abdominal pain, bloody diarrhea, constipation, and fatigue are the main symptoms of the disease, which not only seriously affects the normal working and life of patients, but also increases the risk of secondary infection and colon cancer in patients with long-term recurrence (Terzic et al., 2010; Wang et al., 2016c; Bonovas et al., 2018; Duijvestein et al., 2018).

The occurrence of UC has no gender advantage, and the main age of onset is 30 to 40 years old. In recent years, with the development of urbanization, the incidence and prevalence of UC have been increasing worldwide (Ungaro et al., 2017). Usually, UC has a high incidence and prevalence in Western countries, such as the incidence of UC in Northern Europe is 24.3 per 100,000 persons and its prevalence in Europe is as high as 505 per 100,000 persons. Similarly, the incidence and prevalence of UC in Asian countries (China, Korea, India, Japan, etc.) have also increased significantly. For example, the incidence of UC in Korea is 4.6 per 100,000 persons, and the prevalence of UC in Japan is 121.9 per 100,000 persons (Ng et al., 2016; Ungaro et al., 2017). Because of the unclear etiology and pathogenesis, it is difficult to treat UC in clinic practice. Although aminosalicylic acid, steroids, immunosuppressants and biological agents (e.g., infliximab) can alleviate UC, they have many side effects, including nausea, vomiting, headache, diarrhea, blood dyscrasias, nephrotoxicity, hypokalemia, and increased risk of infection (e.g., herpes zoster, upper respiratory tract infections and urinary tract infections); even some patients do not respond to these drugs (Rosenberg and Peppercorn, 2010; Curro et al., 2017). Therefore, it is particularly urgent to find more reliable and effective prescriptions.

Huang-lian-Jie-du Decoction (HLJDD) is composed of *Coptidis Rhizoma, Scutellariae Radix, Phellodendri Chinensis Cortex* and *Gardeniae Fructus* at the ratio of 3:2:2:3 (Zhou et al., 2017). It is a representative agent for clearing away heat and detoxification in traditional Chinese medicine. The use of this prescription in China can be traced back to more than a thousand years ago (Yu et al., 2017). Currently, the prescription is also widely used in Japan and Korea (Choi et al., 2018; Oshima et al., 2018). According to the theory of traditional Chinese medicine, UC is mainly caused by heat-toxic and damp-heat accumulated in colon tract during the first or acute attack (Zhang, 2012). Therefore, the use of heat-clearing, detoxification, dryness and dampness prescriptions has significant therapeutic effects on UC, such as Rhubarb peony decoction, Huangqin-tang, Pulsatilla decoction, and so on (Wang et al., 2016a; Zou et al., 2016; Luo et al., 2019). In addition, experimental data showed that herbs or drugs with anti-inflammatory and anti-oxidant activities often have certain therapeutic potential for UC (Ding and Wen, 2018; Liu et al., 2018; Saber et al., 2019). Studies have shown that HLJDD has anti-inflammatory, antipyretic, antioxidant, gastrointestinal mucosal protection, etc. properties, which can be used to treat sepsis, gastrointestinal diseases, Alzheimer’s, and other diseases (Miura et al., 2007; Xu et al., 2017; Choi et al., 2018; Sun et al., 2018; Wei et al., 2018). More importantly, in recent years, there have been some cases of clinical application of HLJDD in the treatment of UC in China (Zhang, 2012; Li, 2017). However, the therapeutic effect of HLJDD is not scientifically evaluated and its anti-ulcer mechanism is still unclear.

In view of this, we evaluated the therapeutic effect of HLJDD on DSS-induced UC in mice. In addition, the anti-ulcer mechanism of HLJDD was also explored through mucosal protection and regulation of NF-κB and Nrf2 signaling pathways.

## Materials and Methods

### Animals

Male BABL/c mice (6–8 weeks old, 18–20 g) were purchased from the Animal Center of the Lanzhou Veterinary Research Institute of the Chinese Academy of Agricultural Sciences [SCXK (Gan) 2013-0001]. During the experiment, the mice were housed in an 12 h dark/light circulating environment of room temperature 23 ± 2°C, relative humidity of 55 ± 5%, and free access to standard diet and purified water.

Animal welfare and experimental procedures were carried out in strict accordance with the “Guidelines for the Management and Use of Laboratory Animals” (Ministry of Science and Technology of China, 2006) and approved by the Animal Ethics Committee of Gansu Agricultural University and the Animal Protection and Utilization Committee.

### Materials

*Coptidis Rhizoma*, *Scutellariae Radix*, *Phellodendri Chinensis Cortex*, and *Gardeniae Fructus* were obtained from Lanzhou Yellow River Medicinal Materials Market (Lanzhou, China) and identified by Professor Yanming Wei (College of Veterinary Medicine, Gansu Agricultural University, Lanzhou, China). Voucher specimens of the above four herbs (NO. GSAU_20180501-20180504) were stored in the herbarium center of Gansu Agricultural University. Standard references: berberine (CAS: 2086-83-1), baicalin (CAS: 21967-41-9), geniposide (CAS: 24512-63-8), phellodendrine (CAS: 6873-13-8), chlorogenic acid (CAS: 327-97-9), oroxylin A (CAS: 480-11-5), wogonin (CAS: 632-85-9), magnoflorine (CAS: 2141-09-5), palmatine (CAS: 3486-67-7), wogonoside (CAS: 51059-44-0), coptisine (CAS: 3486-66-6), epiberberine (CAS: 6873-9-2), and jatrorrhizine (CAS: 3621-38-3) were purchased from Nanjing Yuanzhi Biotechnology Co., Ltd (Nanjing, China). The purities of all the above standard products are greater than 98%. Chromatographic grade methanol was obtained from Sigma-Aldrich (St. Louis, MO, USA). Purified water was purchased from Hangzhou Wahaha Group Co., Ltd (Hangzhou, China). Dextran sodium sulfate (DSS) (MW: 36000-50000) was purchased from MP Biomedicals, LLC (Solon, OH, USA). Sulfasalazine (SASP) was purchased from Shanghai Xinyi Tianping Pharmaceutical Co., Ltd (Shanghai, China).

Primary rabbit monoclonal antibodies against NF-κB p65 (CAS: 8242), p-IκBα (CAS: 2859), p-IκKα/β (CAS: 2697), and Keap1 (CAS: 8047) were purchased from Cell Signaling Technology, Inc. (California, USA). Antibody against Nrf2 (CAS: sc-365949) was purchased from Santa Cruz Biotechnology, Inc. (Santa Cruz, CA, USA). Antibodies against Occludin (CAS: 66378-1-Ig), ZO-1 (CAS: 66452-1-Ig), beta Actin (CAS: 60008-1-Ig), GAPDH (CAS: 60004-1-Ig), HRP-conjugated Affinipure Goat Anti-Mouse or Anti-Rabbit IgG (CAS: SA00001-1 and SA00001-15), and CoraLite488-conjugated Affinipure Goat Anti-Mouse or Anti-Rabbit IgG(H+L) (CAS: SA00013-1, SA00013-2) were obtained from Proteintech Group, Inc. (Wuhan, China).

### Preparation of Prescription and HPLC Samples

*Coptidis Rhizoma*, *Scutellariae Radix*, *Phellodendri Chinensis Cortex*, and *Gardeniae Fructus* were mixed in a ratio of 3:2:2:3, soaked in 10 times (v/w) pure water for 1 h, then boiled for 1.5 h and filtered. Eight times (v/w) purified water was added to the residue and was boiled for another 1.5 h, then filtered. The two filtrates were merged and evaporated with rotary evaporation under vacuum at 60°C. After further freeze-drying, HLJDD powder was obtained (yield: 23.2%).

For High Performance Liquid Chromatography (HPLC) analysis, 10 mg HLJDD powder was ultrasonically extracted with 25 ml 30% methanol for 15 min and filtered through a 0.22 µm filter before HPLC analysis. At the same time, the 13 standards were accurately weighed, mixed, and dissolved in methanol to prepare a mixed standard solution, which was diluted before HPLC analysis to establish a standard curve.

*In vivo* treatment, HLJDD powder was added into 0.5% Carboxymethylcellulose sodium (CMC-Na) solution to prepare suspensions with concentrations of 0.92 g/ml, 0.46 g/ml, and 0.23 g/ml (calculating with raw herbs), respectively. The SASP suspension with a concentration of 0.045 g/ml was prepared with 0.5% CMC-Na solution. All the above solutions were stored at 4°C before the experiment.

### High Performance Liquid Chromatography (HPLC) Analysis

Agilent 1260 HPLC (Agilent Technologies, Santa, Clara, CA, USA) equipped with Zorbax Eclipse Plus C18 column (4.6 × 250mm, 5 um) was used for HPLC analysis. Chromatographic separations were performed at 30°C with flow rate of 1.0 mL/min. The injection volume was 20 µL, and the ultraviolet detection wavelength was set as 265 nm. The mobile phase consisted of methanol (A) and water (B) with 1% formic acid (v/v). The gradient elution conditions of the mobile phase A were: 0–10 min, 10%; 10–12 min, 10–15%; 12–14 min, 15–20%; 14–16 min, 20–30%; 16–17 min, 30–31%; 17–21 min, 31–31.4%; 21–30 min, 31.4–35%; 30–35 min, 35–47%; 35–40 min, 47–50%; 40–50 min, 50–65%; 50–60 min, 65–95%.

### Animal Model Preparation and Treatment Procedure

After one week of adaptive feeding, BABL/c mice were randomly divided into six groups, eight mice in each group, namely: normal control (NC), model, sulfasalazine (SASP, 0.45 g/kg), HLJDD high dose (HLJDD-H, 9.2 g/kg), HLJDD medium dose (HLJDD-M, 4.6 g/kg), and HLJDD low dose (HLJDD-L, 2.3 g/kg) groups. During the experiment periods, except for the NC group, the other experimental groups were given 3.5% DSS (w/v) drinking water for 7 consecutive days to induce acute UC model in mice. Fresh DSS water was replaced every morning. At the same time of modeling, each treatment group was given corresponding therapeutic drugs orally at 8:00–10:00 am, once a day for 7 consecutive days. The NC group and the model group were given the same amount of 0.5% CMC-Na solution. The volume of intragastric administration was 0.1 ml/10g body weight.

In the present study, we used the equivalent dose of human clinical dose of HLJDD as the medium dose of HLJDD in mice (HLJDD human clinical dose: 30 g/day, adult weight 60 kg, according to the equivalent body surface area, the dosage of mice were: 30 g ÷ 60 kg × 9.1 ≈ 4.6 g/kg). HLJDD-H was twice the dose of HLJDD-M, and HLJDD-L was 0.5 times the dose of HLJDD-M.

### Assessment of Disease Activity Index (DAI)

The body weight changes, stool consistency, and gross bleeding of each mice were recorded daily during the experiment period, and the Cooper method was slightly modified to quantify the score (Cooper et al., 1993). Scoring criteria were as follows: body weight loss (0: none, 1: 1–5%, 2: 5–10%, 3: 10–20%, 4: > 20%); stool consistency (0: normal, 1: formed feces but easily adhere, 2: semi-formed/soft feces, 3: slurry stool but not adherent to the anus, 4: diarrhea and adherence to the anus); blood in the stool (0: occult blood test negative, 1: weak positive detection of occult blood, 2: occult blood test positive, 3: occult blood test strong positive, 4: gross bleeding). DAI was defined as the sum of the scores of the above three parameters.

### Sample Collection and Macroscopic Assessment of Colitis

At the end of the experiment, mice were anesthetized by intraperitoneal injection of 10% chloral hydrate. Blood was collected from the abdominal aorta (heparin sodium anticoagulation). The plasma was separated by centrifugation at room temperature of 3000 rpm/min for 10 minutes and stored at –80°C for further detection. Further, the mice were sacrificed by cervical dislocation, and the colorectal of the mice were collected. The distance from anus to ileocecal junction was measured. Then the colorectal of three mice in each group were randomly selected and fixed in 10% neutral formalin solution for histopathological examination. The remaining colorectal of the five mice were placed on ice to remove mesentery and adipose tissue and was dissected longitudinally along the mesentery side. According to the scoring system described by Cooper HS, et al., the damage of intestinal mucosa was quantified with macroscopic score (Cooper et al., 1993). The scoring criteria were summarized as following: 0 (normal intestinal mucosa), 1 (mucosa congestion without ulcer lesions and bleeding), 2 (sporadic mucosal ulcer or slight bleeding), 3 (extensive ulcer necrosis or adhesion of intestinal mucosa and bleeding), 4 (severe bleeding and megacolon or stenosis or perforation). After scoring, the intestinal contents were washed with PBS solution (PH = 7.4) and dried with filter paper. Then, the intestinal tissues were frozen by liquid nitrogen and stored at –80°C for further analysis.

### Histopathological Assessment

Colon tissue was fixed in 10% neutral formalin, then embedded in paraffin and cut into 5-µm thick sections. PAS staining and hematoxylin eosin (HE) staining were performed. Pathological photographs were captured by Leica DFC microphotography system. Referring to the scoring system described previously by Ding and Wen, 2018, histopathological score was used to quantify the extent of intestinal injury, including the degree of intestinal epithelial cell injury and the degree of inflammatory infiltration. Briefly, the scoring criteria were summarized as follows: 0 (normal morphology and no inflammation), 1 (loss of goblet cell loss and mild inflammatory infiltration), 2 (large area of goblet cell loss and moderate inflammatory infiltration), 3 (crypt loss and extensive inflammatory infiltration of the mucosal muscular layer with mucosal edema and thickening), 4 (large area of crypt loss and extensive inflammatory infiltration of submucosa layer).

### Detection of Myeloperoxidase (MPO) Activity in Colon Tissue and Plasma

Half of the colon tissues were homogenized and the supernatant were collected. Partial supernatant was used to detect protein concentration using BCA protein assay kit (Solarbio, Beijing, China). Then, the MPO activity in plasma and colon homogenate supernatant of mice were detected according to the instructions of MPO assay kit (Nanjing jiancheng, Nanjing, China).

### Assessment of Plasma Cytokines and Antioxidant Parameters

Plasma cytokines contents of TNF-α, IL-1β, and IL-10 were measured by ELISA using mouse TNF-α, IL-1β, and IL-10 assay kits (Neobioscience, Shenzhen, China), respectively, according to the manufacturer’s protocols. In addition, nitric oxide (NO), malondialdehyde (MDA), glutathione (GSH), and superoxide dismutase (SOD) assay kits (Nanjing jiancheng, Nanjing, China) were used to investigate NO, MDA, GSH, and SOD levels in colon homogenate.

### Western Blot Analysis

Colon tissues were cut into pieces and lysed in RIPA buffer (Solarbio, Beijing, China) with protein phosphatase inhibitor (Solarbio), and total proteins were extracted according to the manufacturer’s protocols. Then, the protein concentration was measured using BCA protein assay kit (Solarbio). 50 µg of total protein was separated by 10% SDS-polyacrylamide gel electrophoresis (SDS-PAGE) and then transferred onto 0.22 µm polyvinylidene fluoride (PVDF) membrane (Millipore, MA, USA). After washing twice in 1 × TBS buffer, the membranes were blocked with 1 × TBST containing 5% nonfat milk (or 5% BSA for phospho-epitope antibodies) for 1 h at room temperature. Then, the membranes were incubated with primary antibodies overnight at 4°C. The primary antibodies and its dilution concentration were as follows: NF-κB-p65 (1:1000), p-IκBα (1:1000), p-IκKα/β (1:1000), Keap1 (1:1000), Nrf2 (1:1000), Occludin (1:4000), ZO-1 (1:4000), beta Actin (1:5000), and GAPDH (1:5000). Subsequently, the membranes were washed with 1 × TBST and incubated with secondary antibodies: HRP-conjugated Affinipure Goat Anti-Mouse or Anti-Rabbit IgG (1:5000) for 1 h at room temperature. After the membranes were washed again by 1 × TBST buffer, the proteins were visualized using enhanced chemiluminescence (Vazyme, Nanjing, China). The Amersham Imager 600 chemiluminometer (GE Healthcare Bio-Sciences AB, Sweden) was used for signal acquisition and photographing. ImageJ software was used to analyze the gray value of protein bands.

### Immunofluorescence Assay

Paraffin-embedded colonic tissues were cut into 3 µm thick sections. After the sections were dewaxed with xylene and a gradient alcohol solution (100%, 95%, 90%, 80%), sodium citrate solution was used for tissue antigen retrieval (120°C for 10 min). The sections were washed with PBS solution (PH 7.2) and blocked with 5% BSA for 1 h at room temperature. Then the primary antibody (ZO-1, 1:400 dilution; Occludin, 1:200 dilution; NF-κB p65, 1:400 dilution; Nrf2, 1:400 dilution) was added and incubated overnight at 4°C. The sections were washed with PBS solution and then incubated with CoraLite488-conjugated secondary antibody (1:500) for 1 h at room temperature. Subsequently, the sections were washed with PBS solution again and then mounting medium (with DAPI) (Solarbio) was added for sealing. Finally, DeltaVisionTM Ultra (GE Healthcare Bio-Sciences Corp., Marlborough, USA) was used to collect photographs.

### Statistical Analysis

All data were expressed as mean ± standard deviation (SD). One-way analysis of variance (ANOVA) followed by a Tukey-Kramer multiple comparison test was used for statistical comparison between parametric data. Scoring data were analyzed with nonparametric test (Mann-Whitney U test). GraphPad Prism software version 6.0 (GraphPad Software Inc., La Jolla, CA, USA) was used for statistical analysis and plotting. In all experiments, p < 0.05 was considered statistically significant.

## Results

### HPLC Detection of HLJDD Active Ingredients

**Figure 1:**
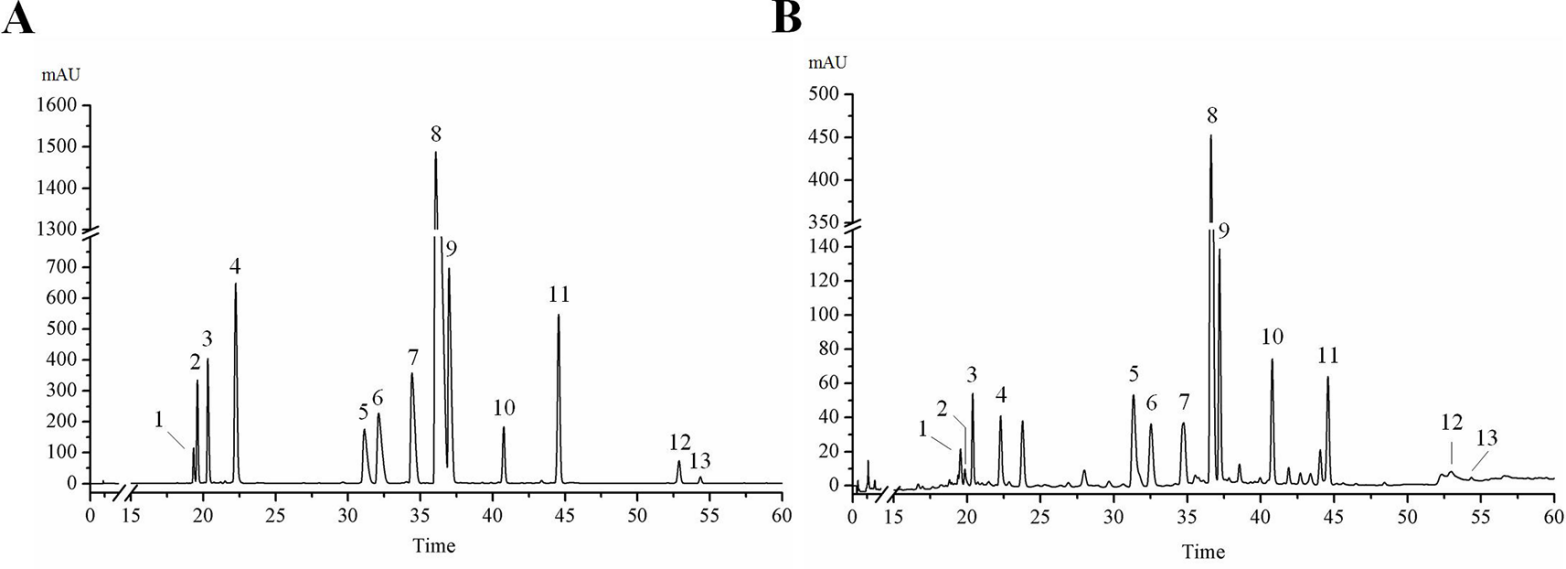
HPLC chromatogram of 13 active ingredients in HLJDD. **(A)** Mixed standards, **(B)** HLJDD sample. Compounds: phellodendrine (1), chlorogenic acid (2), magnoflorine (3), geniposide (4), coptisine (5), epiberberine (6), jatrorrhizine (7), berberine (8), palmatine (9), baicalin (10), wogonoside (11), wogonin (12), oroxylin A (13).

HPLC analysis was performed according to the chromatographic conditions described in Section 2.3. As can be seen from **Figure 1** (**Data S1**), the baseline of mixed standard solution and HLJDD sample were stable, and the chromatographic peaks of each active ingredient were separated perfectly. The results of precision, stability, and repeatability test showed that the relative standard deviation (RSD) values of retention time and peak area of each active ingredient were less than 0.1% and 5%, respectively (**Tables S1–3**). In addition, the results of the sample recovery test showed that the RSD of recovery of 13 active ingredients were less than 5% (**Table S4**). These results indicate that the HPLC method we have established was stable and reliable, and can be used for subsequent sample detection.

**Figure 2:**
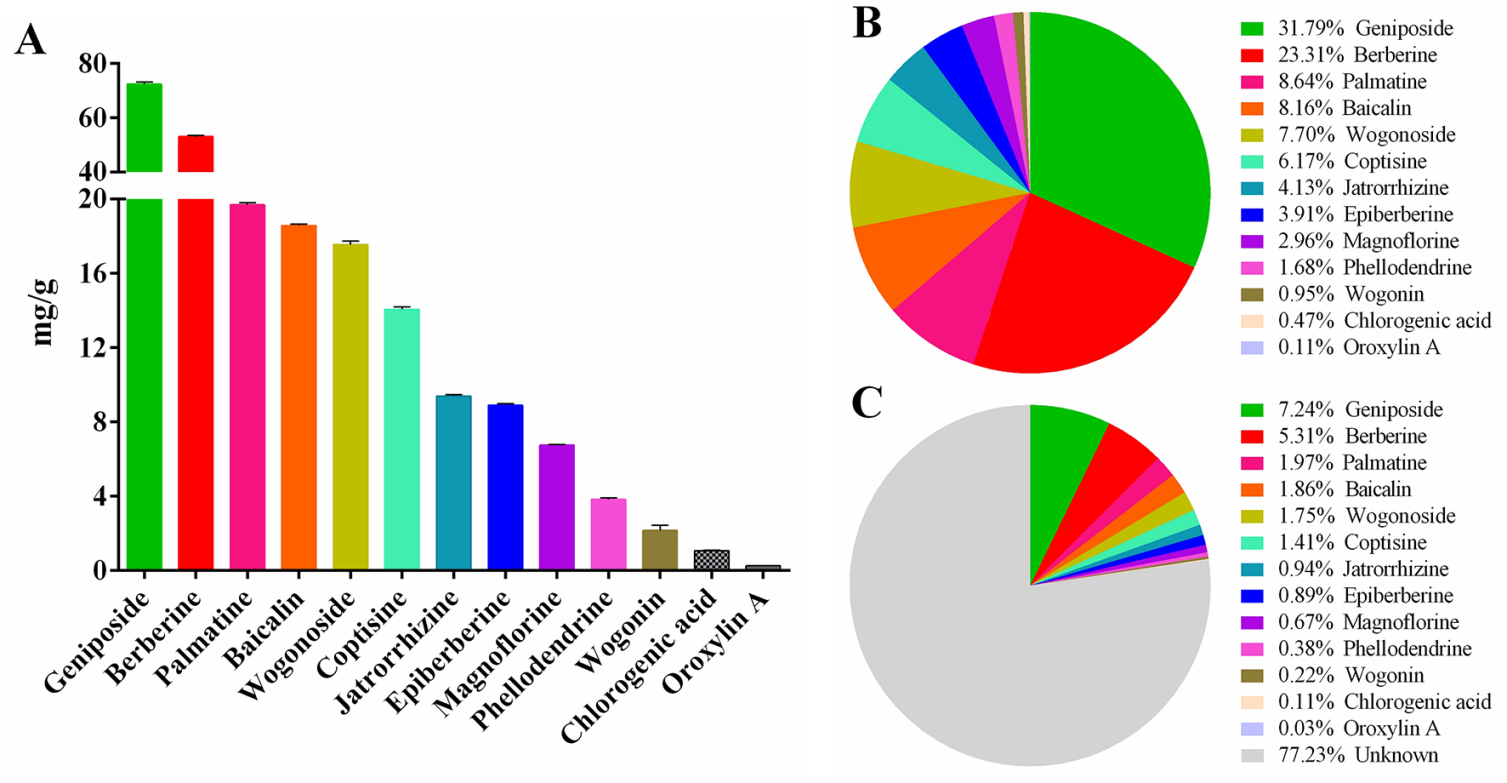
Contents of 13 active ingredients in HLJDD. **(A)** The content of 13 active ingredients in HLJDD. **(B)** The percentage of each active ingredient in the total 13 active ingredients. **(C)** The percentage of 13 active ingredients in HLJDD.

The results of HPLC showed that the linear relationship of each active ingredient was good within the corresponding detection concentration range (**Table 1**). HLJDD has high content of geniposide, berberine, palmatine, baicalin, wogonoside, coptisine, jatrorrhizine, and epiberberine, accounting for 93.81% of the total content of 13 active ingredients (**Figures 2A, B** and **Table S5**), which are the main active ingredients of HLJDD. However, these 13 active ingredients only account for 22.77% of the total HLJDD (**Figure 2C**), indicating that there are still many unknown components in HLJDD.

**Table 1:** Linear regression equations of 13 active ingredients in HLJDD

Ingredients	Regression equation	Correlation coefficient	Linear range (μg/ml)	Herb source
Phellodendrine	y = 4.2829x +0.1766	0.9996	0.0358 - 11.440	*Coptidis Rhizoma & Phellodendri Chinrnsis Cortex*
Chlorogenic acid	y = 18.962x -6.0744	0.9997	1.1250 - 36.000	*Coptidis Rhizoma & Gardeniae Fructus*
Magnoflorine	y = 44.068x -1.0867	0.9999	0.0405 - 12.960	*Coptidis Rhizoma & Phellodendri Chinrnsis Cortex*
Geniposide	y = 5.1308x +0.4947	0.9958	0.4690 - 300.00	*Gardeniae Fructus*
Coptisine	y = 59.547x -30.839	0.9987	0.1313 - 42.000	*Coptidis Rhizoma & Scutellariae Radix & Phellodendri Chinrnsis Cortex*
Epiberberine	y = 56.062x -7.1212	0.9992	0.0359 - 11.480	*Coptidis Rhizoma & Scutellariae Radix*
Jatrorrhizine	y = 58.775x -2.6186	0.9998	0.0480 - 15.360	*Coptidis Rhizoma & Scutellariae Radix & Phellodendri Chinrnsis Cortex*
Berberine	y = 90.337x -204.46	0.9995	1.5000 - 960.00	*Coptidis Rhizoma & Phellodendri Chinrnsis Cortex*
Palmatine	y = 59.303x -257.76	0.9995	0.7297 - 467.00	*Coptidis Rhizoma & Phellodendri Chinrnsis Cortex*
Baicalin	y = 33.606x -15.172	0.9949	0.1150 - 73.800	*Scutellariae Radix*
Wogonoside	y = 45.876x +4.4079	0.9997	0.0657 - 42.000	*Scutellariae Radix*
Wogonin	y = 38.580x -2.6273	0.9994	0.0328 - 21.000	*Scutellariae Radix*
Oroxylin A	y = 27.353x -2.7089	0.9993	0.0422 - 27.000	*Scutellariae Radix*

### HLJDD Alleviates DSS-Induced UC Symptoms

**Figure 3:**
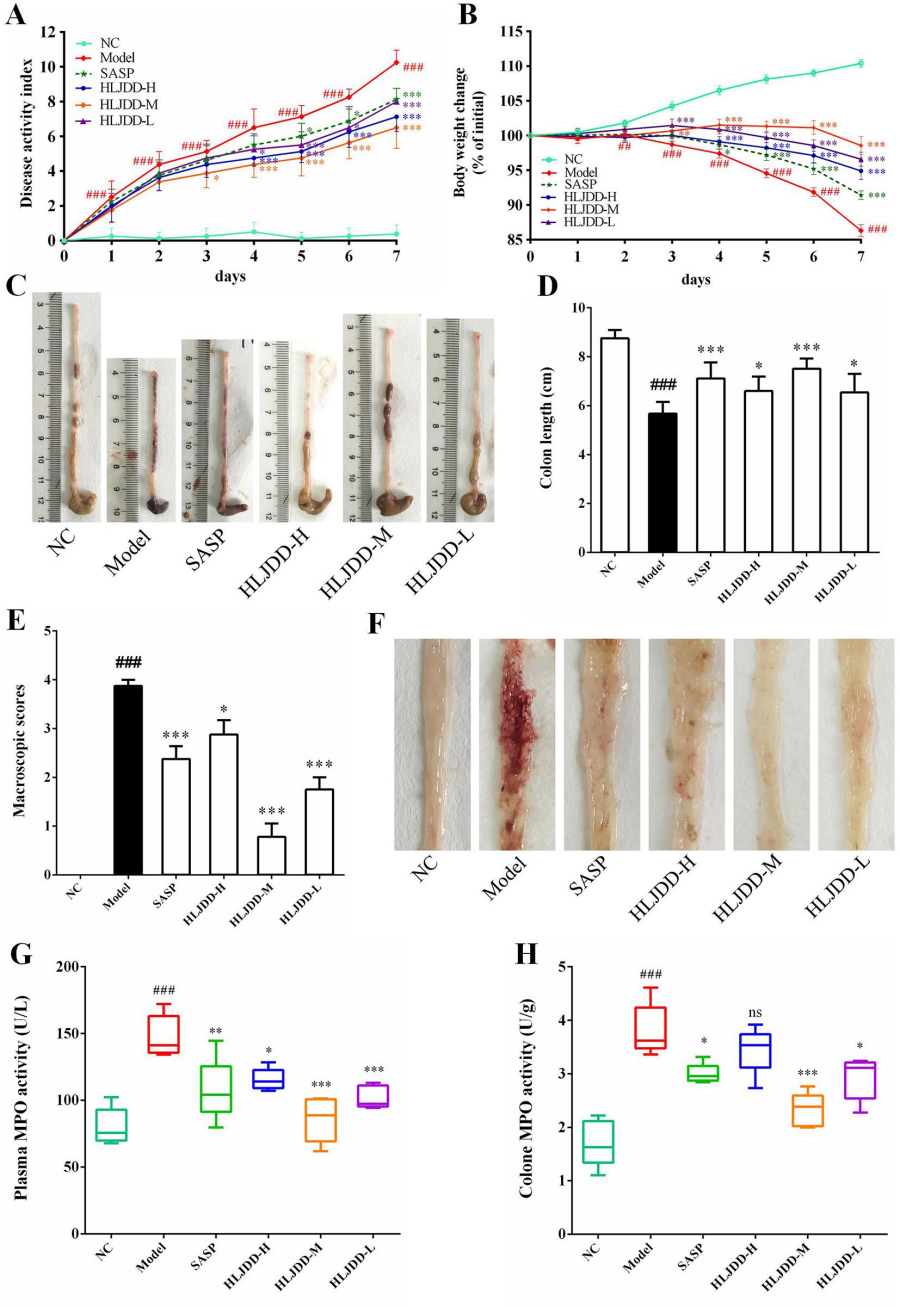
HLJDD intervention relieves DSS-induced UC in mice. Disease activity index **(A)** and body weight change **(B)** of mice during experiment period. HLJDD alleviates DSS-caused colon length reduction **(C**, **D)** and intestinal injury **(E**, **F)**. HLJDD treatment reduces MPO activity in plasma **(G)** and colon tissues **(H)** of UC mice. Data are expressed as the mean ± SD, except for MPO activity analysis n = 5 per group, the remaining analysis n = 8 per group. ^##^*p* < 0.01, ^###^*p* < 0.001 vs. NC group; *p < 0.05, **p < .01, *** < *p* 0.001 vs. model group. ns, no significant.

In this study, we used DSS to establish an acute UC model in BABL/c mice. Our results showed that with continuous intake of 3.5% DSS drinking water, the mice in the model group gradually developed clinical symptoms such as body weight loss, depression, lethargy, bloody stool, and diarrhea, and their disease activity index (DAI) was significantly higher than that in the NC group (**Figures 3A, B** and **Table S6**, **S7**). In addition, the colon length of the model group was significantly shortened (**Figures 3C, D**), and a large amount of hemorrhage, ulcerative lesions, and mucosal exfoliation were observed by colonic necropsy (**Figure 3F**). Compared with the NC group, the colon macroscopic score was significantly increased (*p* < 0.001) (**Figure 3E**). Furthermore, plasma and colon tissue myeloperoxidase (MPO) activity assay showed that, compared with the NC group, the plasma and colon MPO activity was significantly increased (*p* < 0.001) (**Figures 3G, H**). These results showed that the acute UC model of mice was successfully established.

Different doses of HLJDD were given for intervention during the trial. The results showed that compared with the model group, HLJDD intervention (especially HLJDD-M) could significantly reduce DAI in mice (*p* < 0.001), inhibit weight loss and colon shortening (*p* < 0.05), significantly alleviate visible damage of intestinal caused by DSS (*p* < 0.05), and significantly reduce MPO activity in plasma and colon of mice (*p* < 0.05) (**Figure 3**) (**Data S2**, **S3**). These results indicate that HLJDD has the potential to treat UC.

### Colon Histopathological Examination

**Figure 4:**
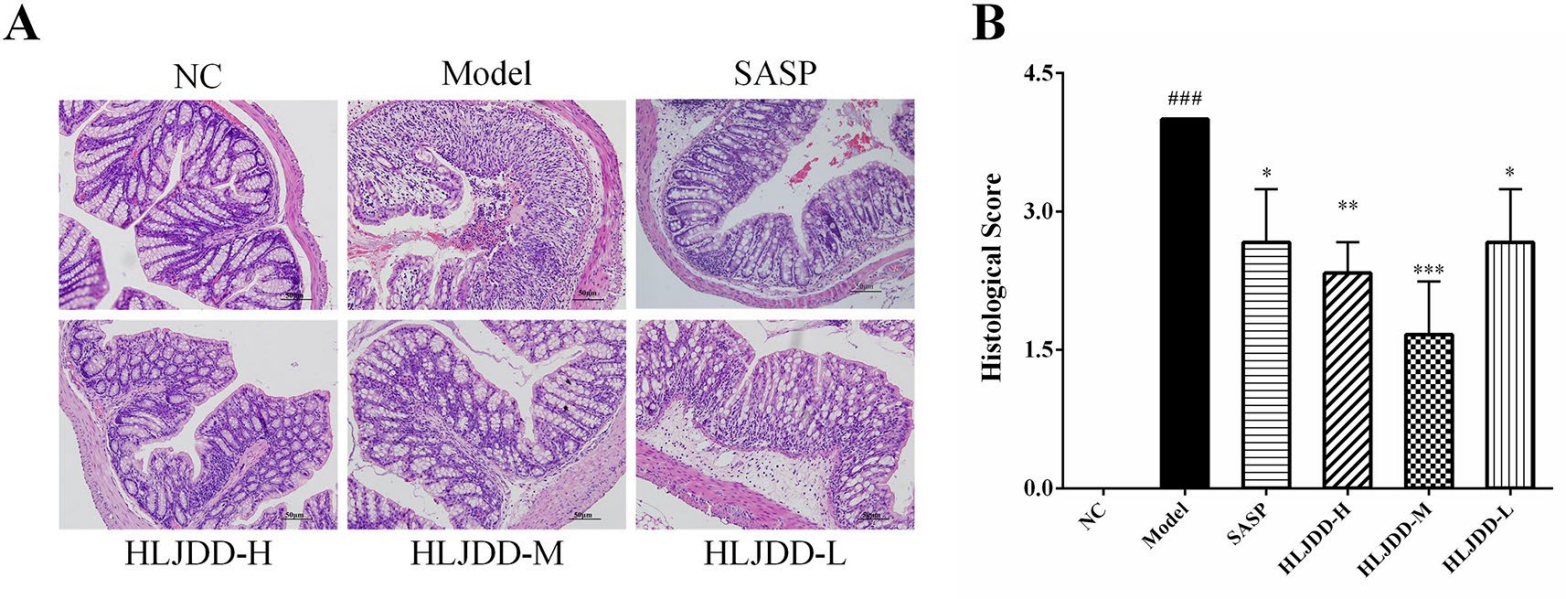
HLJDD attenuates colonic pathological damage in acute UC mice. **(A)** Representative images showing colon pathologic damages with hematoxylin and eosin (H&E) staining, (200 × magnification). **(B)** Histological scores of colons. Data are expressed as the mean ± SD, n = 3 pre group. ^###^*p* < 0.001 vs. NC group; *p < 0.05, **p < 0.01, ***p < 0.001 vs. model group.

The results of histopathological examination of colon tissue showed that the colonic mucosal structure of the model group was seriously damaged, the crypt was extensively deficient, and there were hemorrhage, edema, and massive inflammatory cell infiltration in the mucosa and submucosa layer (**Figure 4A** and **Figure S2**). Compared with the NC group, the histopathological score of colon in the model group increased significantly (*p* < 0.001) (**Figure 4B**). However, the colon structure of mice treated with HLJDD was clearly visible, with mild or moderate inflammatory cell infiltration and edema in the mucosa and submucosa layer (**Figure 4A**). Compared with the model group, different doses of HLJDD treatment could significantly reduce the histopathological score of colon (*p* < 0.05) (**Figure 4B**), indicating that HLJDD could significantly alleviate DSS-induced colon injury.

### Effects of HLJDD on Plasma Cytokines in UC Mice

**Figure 5:**
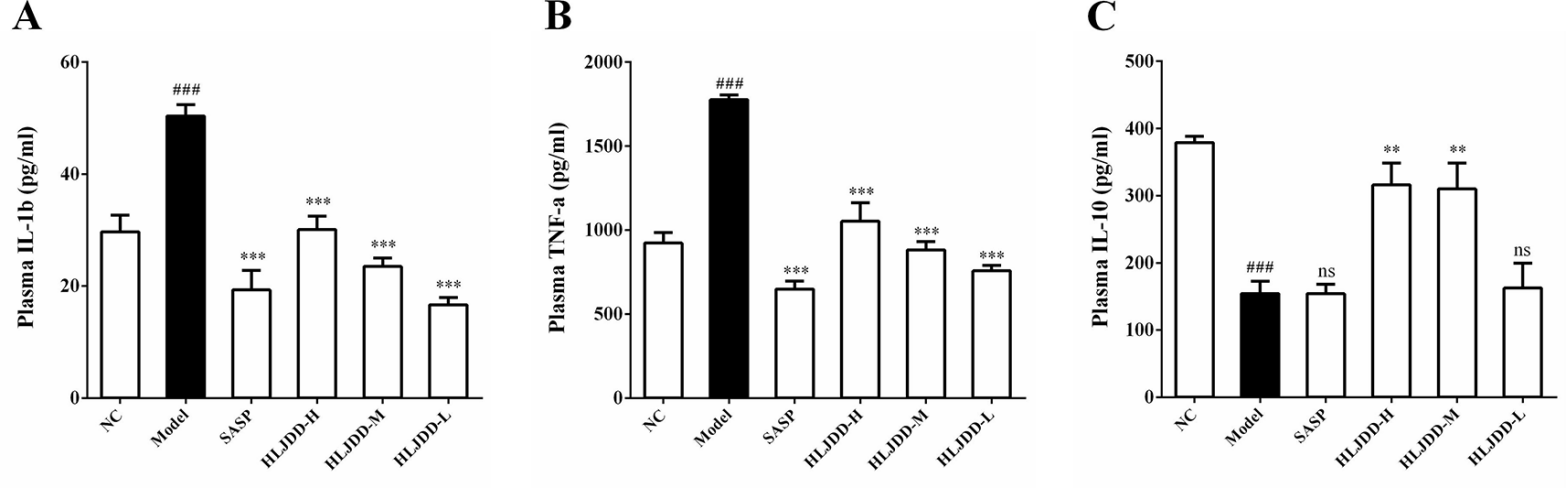
Effects of HLJDD on plasma cytokines in UC mice. Treatment with HLJDD down-regulated plasma IL-1β **(A)**, TNF-α **(B)**, and up-regulated IL-10 **(C)** levels in UC mice. Data are expressed as the mean ± SD, n = 5 per group. ^###^*p* < 0.001 vs. NC group; **p < 0.01, ***p < 0.001 vs. model group. ns: no significant.

Mice plasma were collected and used for cytokine detection. The results showed that compared with the NC group, the plasma levels of IL-1β and TNF-α in the model group were significantly increased (*p* < 0.001) (**Figures 5A, B**), while the levels of IL-10 was significantly decreased (*p* < 0.001) (**Figure 5C**), indicating that the immune function of the model group mice was abnormal and there were significant inflammatory reactions. Compared with the model group, different doses of HLJDD could significantly reduce the levels of IL-1β and TNF-α in plasma of mice (*p* < 0.001), and high/medium doses of HLJDD could significantly increase the levels of IL-10 in plasma (*p* < 0.01) (**Figure 5**) (**Data S4**). These results suggest that the anti-inflammatory effect of HLJDD is partly responsible for the treatment of UC.

### Effects of HLJDD on Colon Oxidative Stress Parameters in UC Mice

**Figure 6:**
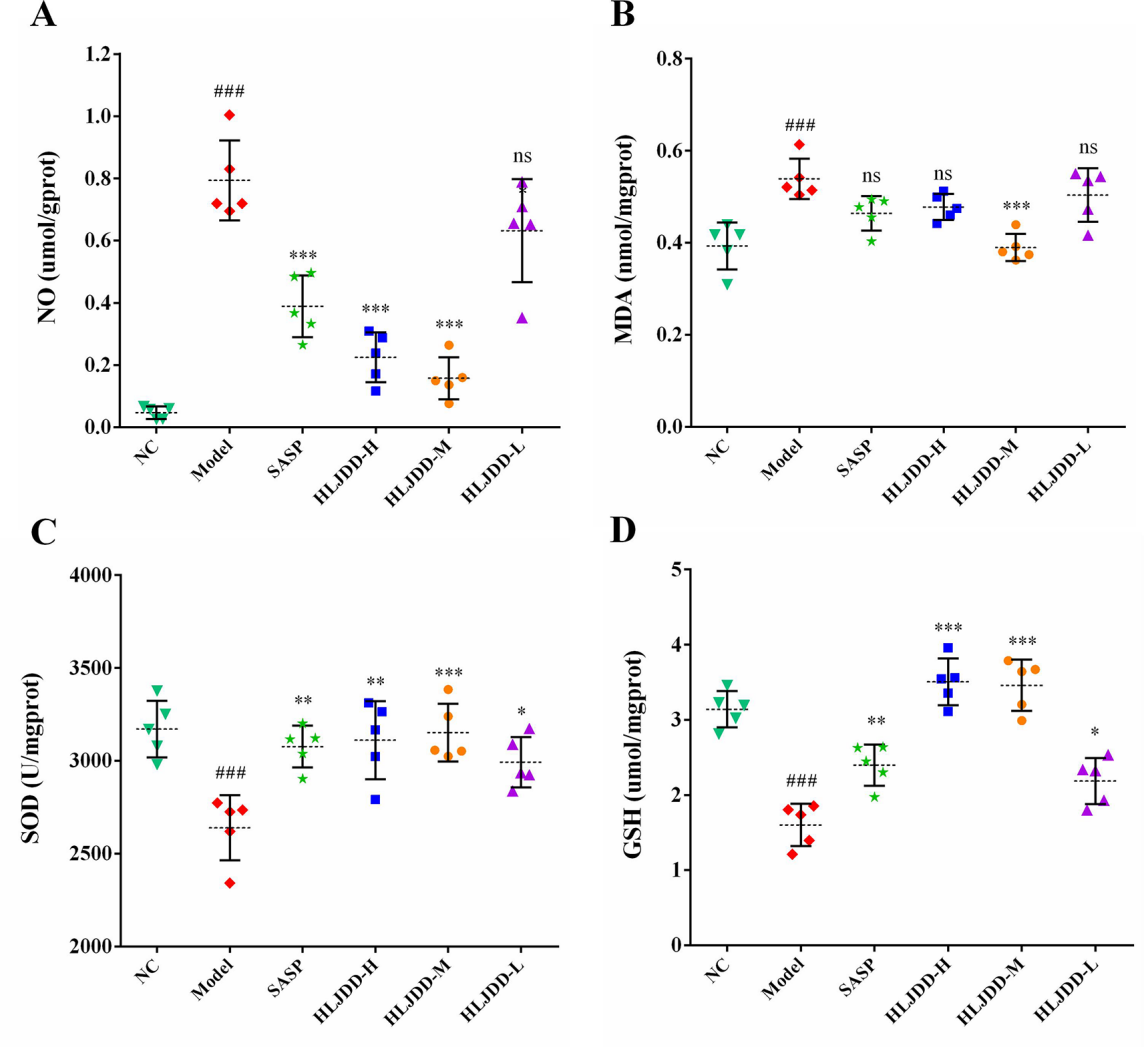
Antioxidant effect of HLJDD on DSS-induced UC. Levels of NO **(A)**, MDA **(B)**, SOD activity **(C)**, and GSH **(D)** in colon homogenate. Data are expressed as the mean ± SD, n = 5 per group. ^###^*p* < 0.001 vs. NC group; *p < 0.05, **p < 0.01, ***p < 0.001 vs. model group. ns, no significant.

The results of antioxidant parameters test showed that the content of NO and MDA in colon homogenate of model group mice were significantly increased (*p* < 0.001) (**Figures 6A, B**), and the content of antioxidant GSH and the activity of SOD antioxidant enzyme were significantly decreased (*p* < 0.001) (**Figures 6C, D**), indicating that the levels of oxidative stress in model group mice were significantly enhanced. Different doses of HLJDD intervention could significantly increase GSH content and SOD activity (*p* < 0.05) and significantly reduce NO content (*p* < 0.05) (**Figures 6A, C, D**). Besides, only the HLJDD-M treatment could significantly reduce the level of MDA (*p* < 0.001) (**Figure 6B**) (**Data S4**). These results indicate that the therapeutic effect of HLJDD on UC is also derived from its antioxidant effect.

### Inhibitory Effect of HLJDD on NF-κB Signaling Pathway in UC Mice

**Figure 7:**
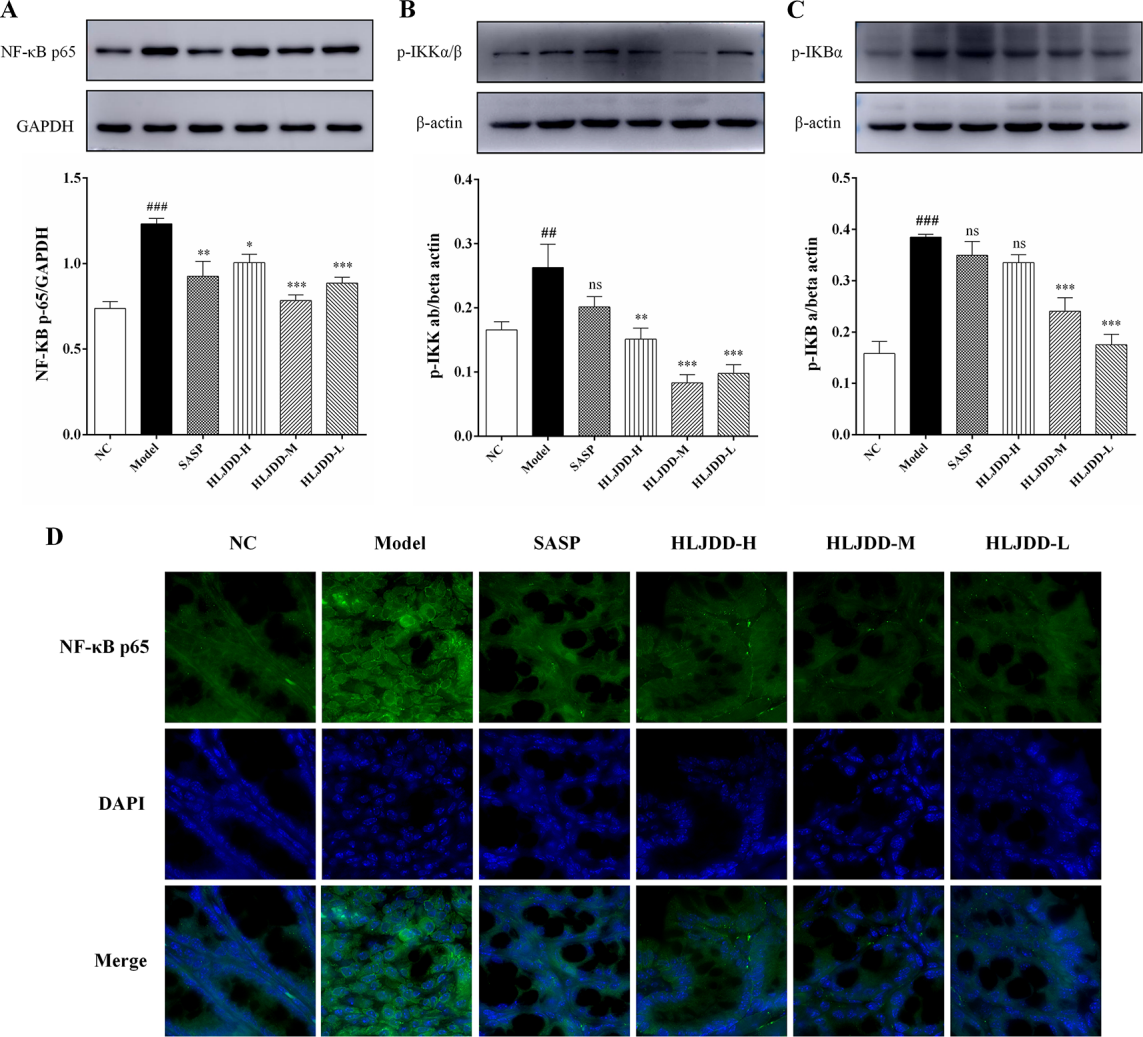
Inhibitory effect of HLJDD on NF-κB signaling pathway in UC mice. Western blot analysis of protein expression levels of NF-κB p65 **(A)**, p-IκKα/β **(B)**, and p-IκBα **(C)** in colon tissue. **(D)** Immunofluorescence analysis of NF-κB p65 (green) in colon mucosa (600 × magnification). DAPI was used for nuclear staining (blue). Data are expressed as the mean ± SD, n = 5 per group. ^##^*p* < 0.01, ^###^*p* < 0.001 vs. NC group; *p < 0.05, **p < 0.01, ***p < 0.001 vs. model group. ns, no significant. (For interpretation of the references to color in this figure legend, the reader is referred to the web version of this article).

Since the nuclear transcription factor kappa B (NF-κB) signaling pathway plays an important role in the inflammatory process, we detected this signaling pathway by western blot. Compared with the NC group, the levels of NF-κB p65, p-IκKα/β, and p-IκBα in the model group mice were significantly increased (*p* < 0.01) (**Figure 7** and **Figure S1**), indicating that the NF-κB signaling pathway in the colon tissue of the model group was activated, which can be used to partially explain the abnormal elevation of inflammatory cytokines in the model group mice. HLJDD treatment could significantly down-regulate the expression levels of NF-κB p65, p-IκKα/β, and p-IκBα (**Figure 7**) (**Data S5**), which reveals that the inhibition of NF-κB signaling pathway is one of its therapeutic mechanisms.

### Activation Effect of HLJDD on Nrf2 Signaling Pathway in UC Mice

**Figure 8:**
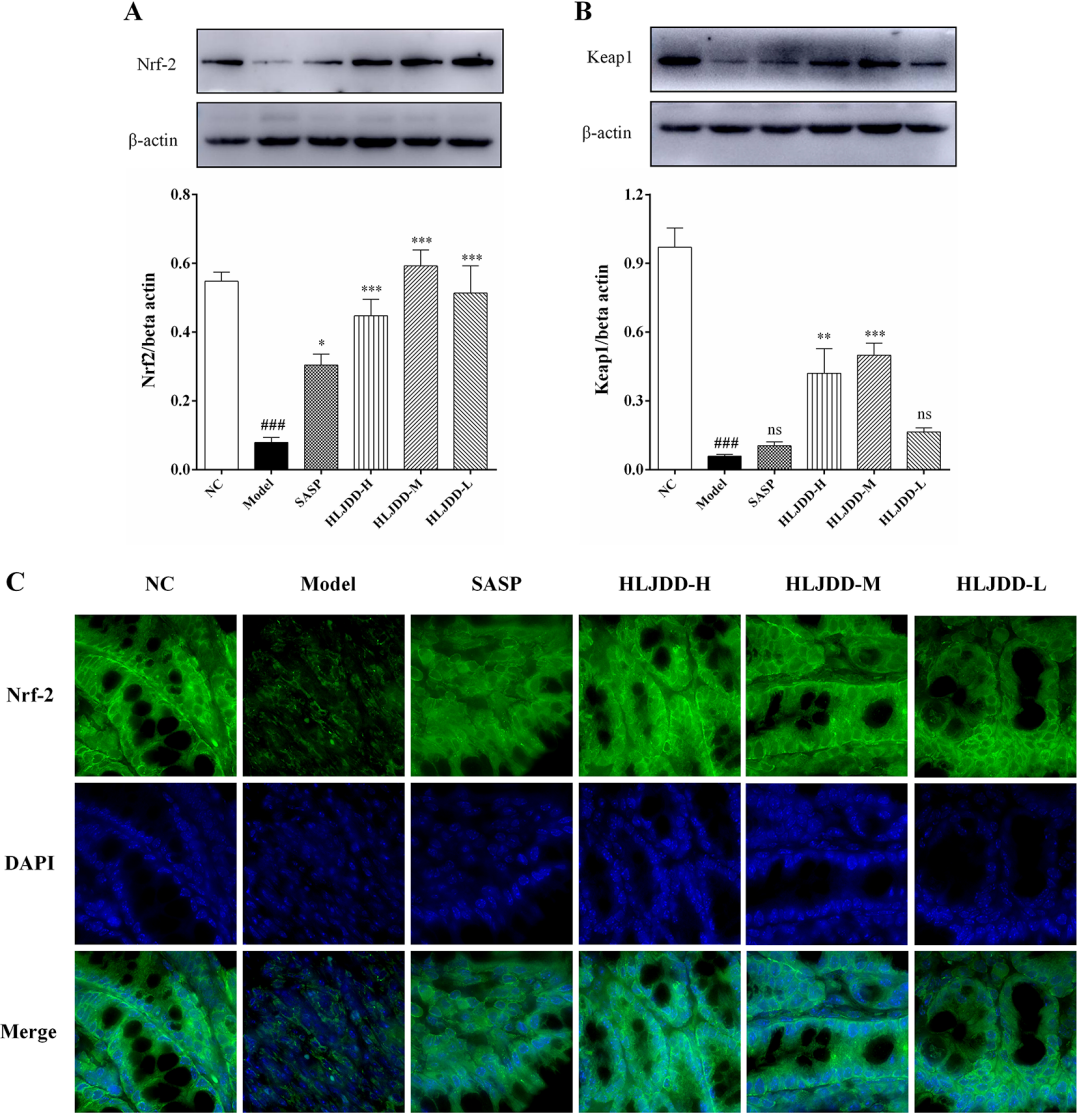
The regulation effect of HLJDD on Nrf2 signaling pathway. HLJDD therapy up-regulates the expression of key proteins Nrf2 **(A)** and Keap1 **(B)** involved in the Nrf2 signaling pathway in colon; **(C)** Immunofluorescence analysis of Nrf2 (green) in colon mucosa (600 × magnification). DAPI was used for nuclear staining (blue). Data are expressed as the mean ± SD, n = 5 per group. ^###^*p* < 0.001 vs. NC group; *p < 0.05, **p < 0.01, ***p < 0.001 vs. model group. ns, no significant. (For interpretation of the references to color in this figure legend, the reader is referred to the web version of this article).

As shown in **Figure 8**, our results showed that compared with the NC group, the levels of nuclear factor erythroid 2-related factor 2 (Nrf2) and Kelch-like ECH-associated protein 1 (Keap1) in the colon tissue of the model group were significantly reduced (*p* < 0.001), indicating that the antioxidant signaling pathway was significantly inhibited in the model group. This is consistent with the results of the antioxidant parameters test (**Figure 6**). HLJDD intervention could significantly up-regulate the Nrf2 and Keap1 protein levels in colon of mice (**Figure 8** and **Figure S1**), (**Data S5**), suggesting that HLJDD treatment could significantly activate Nrf2 signaling pathway.

### The Intestinal Mucosal Protective Effect of HLJDD

**Figure 9:**
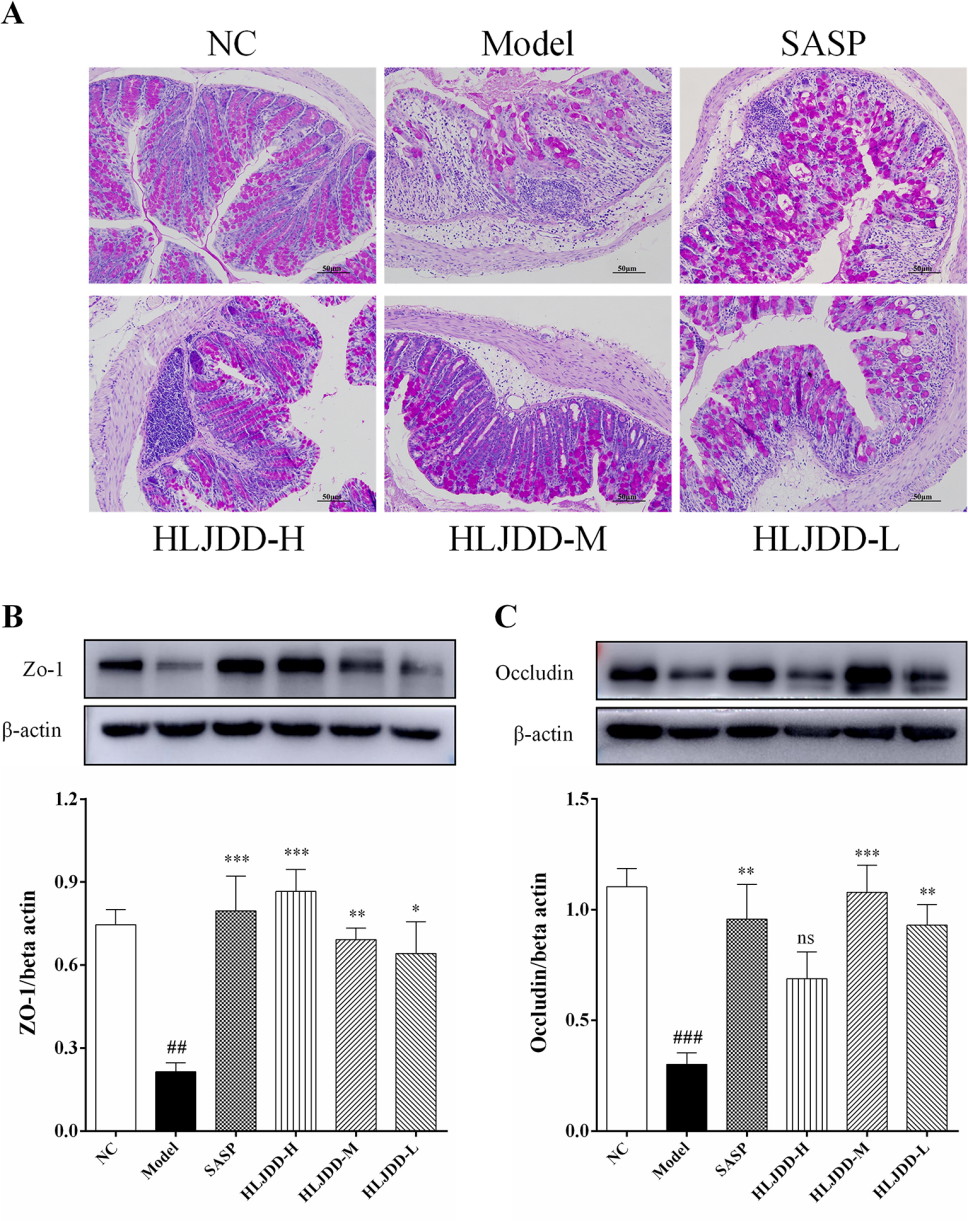
The intestinal mucosal protective effect of HLJDD. **(A)** PAS staining of the colon tissue of different groups (200 × magnification). Western blot analysis of tight junction protein expression levels of ZO-1 **(B)** and Occludin **(C)** in colon tissue. Data are expressed as the mean ± SD, n = 5 per group. ^##^*p* < 0.01, ^###^*p* < 0.001 vs. NC group; *p < 0.05, **p < 0.01, ***p < 0.001 vs. model group. ns, no significant.

**Figure 10:**
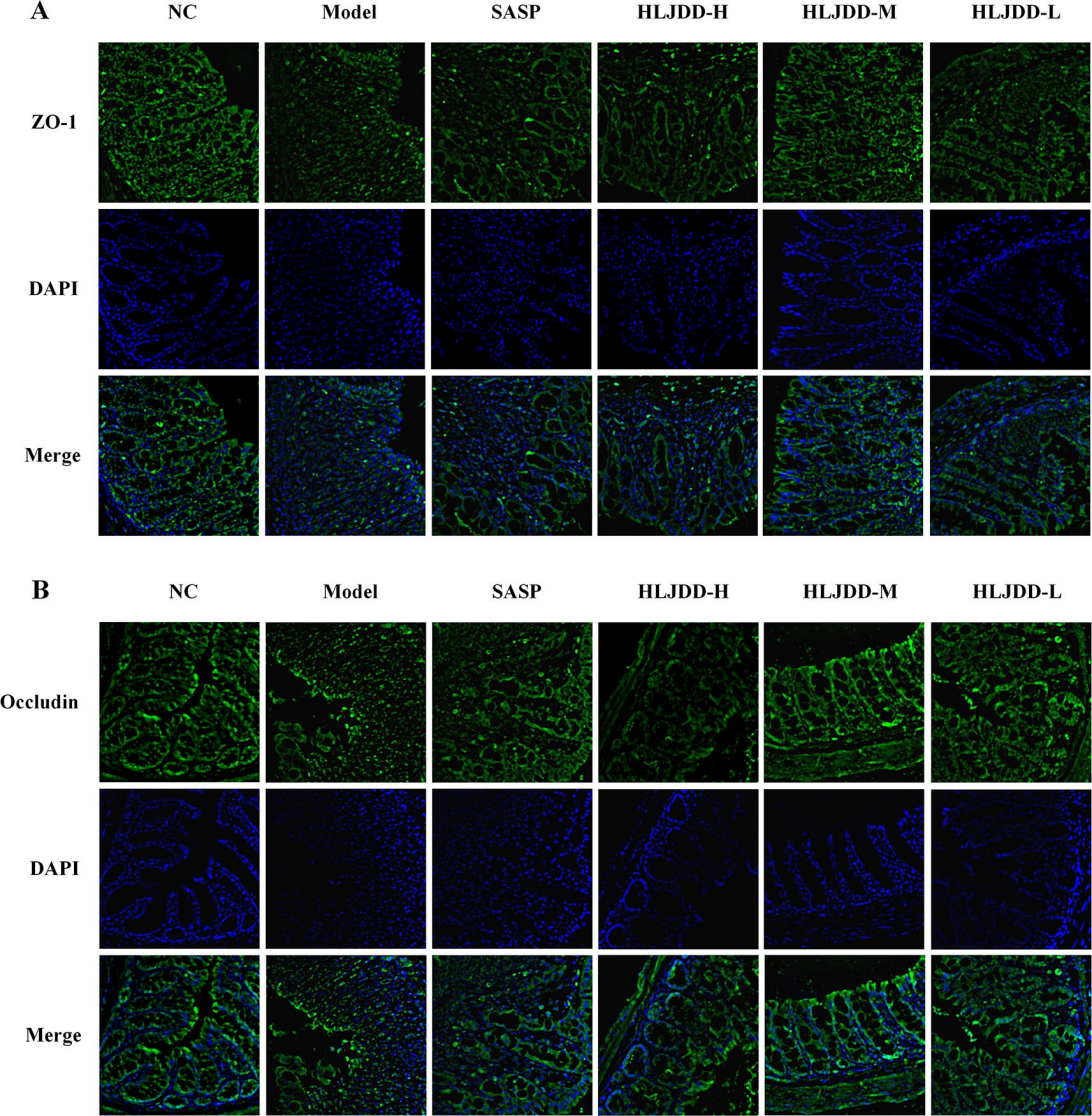
HLJDD reduces DSS-induced destruction of tight junction proteins in colon mucosa of mice. Immunofluorescence analysis of ZO-1 (green) **(A)** and Occludin (green) **(B)** in colon mucosa (200 × magnification). DAPI was used for nuclear staining (blue). (For interpretation of the references to color in this figure legend, the reader is referred to the web version of this article.)

There are a large number of goblet cells in the colon tissue. The mucus protein (a glycogen protein) secreted by goblet cells is an important component of the intestinal mucosal mechanical barrier. Therefore, we tested it by PAS staining. As can be seen from **Figure 9A** and **Figure S3**, compared with the NC group, the colon mucus protein in the model group was significantly reduced, which could be significantly inhibited by HLJDD treatment. Besides, the assay results of tight junction protein [zona occludens protein 1 (ZO-1) and occludin] showed that HLJDD treatment could significantly reverse the decrease of ZO-1 and occludin protein expression in colon tissue induced by DSS (**Figures 9B, C** and **Figure S1**) (**Data S5**). Additionally, we also detected ZO-1 and occludin by tissue immunofluorescence. From **Figure 10**, we can see that HLJDD treatment could significantly increase the expression of ZO-1 and occludin in mouse colon epithelial cells and form a dense fluorescent ring, which more intuitively shows that HLJDD could strengthen the links between epithelial cells. These results suggest that HLJDD has significant intestinal mucosal protective effect, which is another mechanism of HLJDD in the treatment of UC.

## Discussion

**Figure 11:**
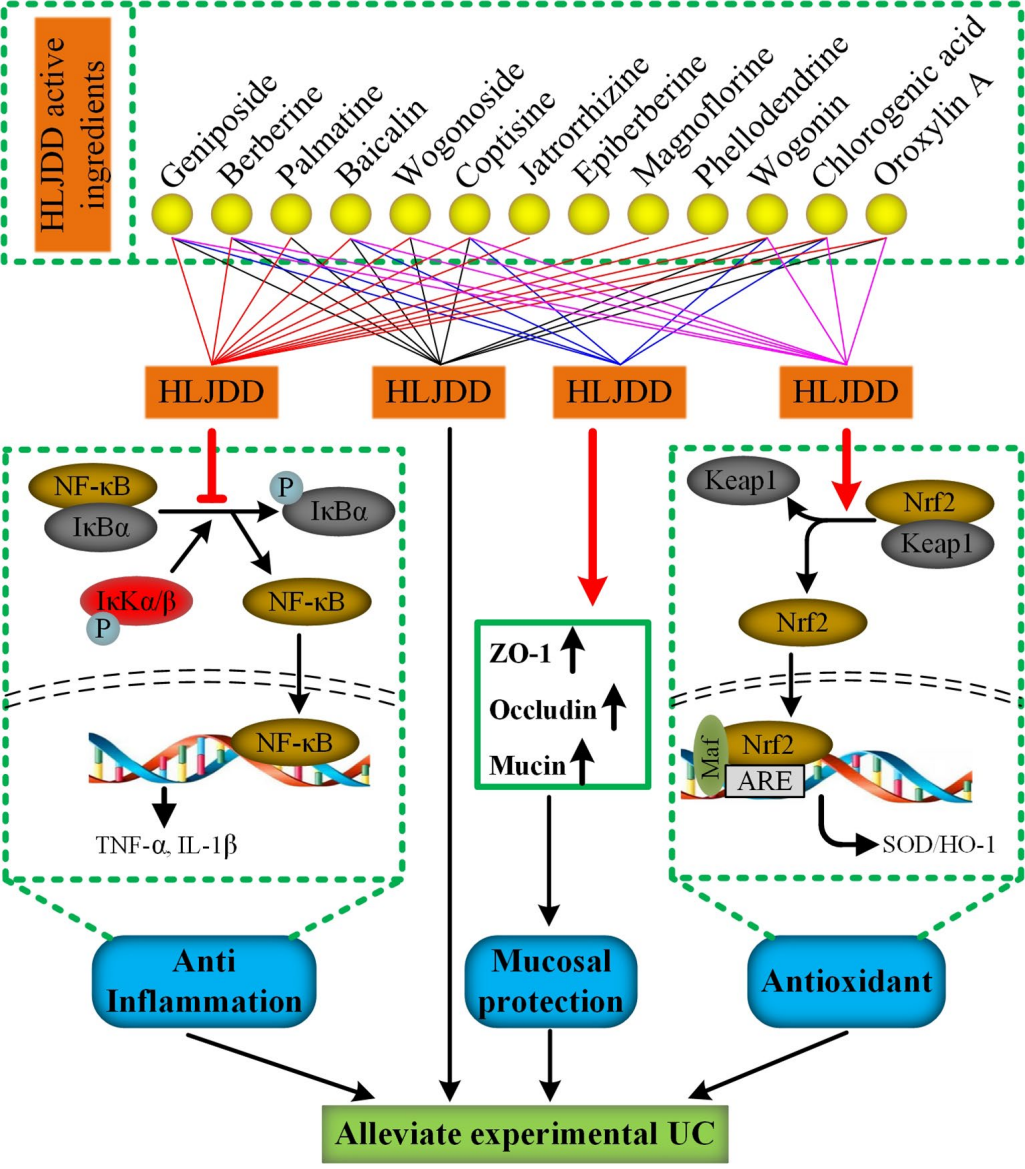
The mechanism of HLJDD alleviate DSS-induced UC. (For interpretation of the references to color in this figure legend, the reader is referred to the web version of this article)

A study 20 years ago reported that HLJDD can be used to treat inflammatory bowel disease. (Zhou and Mineshita, 1999). However, due to the limitation of the scientific technology at that time, its mechanism has not been revealed. Different from previous studies, we used DSS to establish an acute UC model in mice, a classical experimental animal model of UC studies with clinical manifestations or pathological features most similar to human UC (Bamba et al., 2012). In addition, the proportion of HLJDD we use is the traditional proportion, and its application can be traced back to more than a thousand years ago, which is different from previous studies (Miura et al., 2007; Si et al., 2012). At present, the improvement of clinical symptoms and histological recovery are important indicators for evaluating the efficacy of drug therapy for UC (Peyrin-Biroulet et al., 2015). Therefore, in this study, we comprehensively evaluated the therapeutic effect of HLJDD on UC mice by quantifying the clinical manifestations of mice and histopathological examination of colon tissue of mice. More importantly, we have revealed some of its mechanisms (**Figure 11**).

To some extent, IBD is considered to be an autoimmune disease just like rheumatoid arthritis, psoriasis, and systemic lupus erythematosus (Ungaro et al., 2017). Indeed, there are significant inflammatory and immune dysfunctions in IBD patients and colitis animal models, which is manifested by an imbalance in the secretion of pro-inflammatory cytokines and anti-inflammatory cytokines (Soufli et al., 2016). Proinflammatory cytokines such as TNF-α and IL-1β (mainly produced by macrophages) play an active role in the formation of inflammation. They not only promote the expression of adhesion molecules in endothelial cells to assist neutrophil migration, but also increase the phagocytic capacity of macrophages and induce the release of inflammatory mediators (PGE2) to further activate and amplify the cascade of inflammatory signals, which is an important mediator in the early stage of inflammation (Zhang et al., 2019). However, excessive synthesis secretion of pro-inflammatory cytokines such as TNF-α and IL-1β often causes certain damage to the body (Scott et al., 2010; Soufli et al., 2016). Previous studies have shown that TNF-α levels are significantly increased in IBD patients (Murch et al., 1993; Billmeier et al., 2016). The treatment of IBD patients with TNF-α monoclonal antibody (such as infliximab and golomumab) could effectively alleviate the disease (Rutgeerts et al., 2005; Sandborn et al., 2014; Iwanczak et al., 2017). In addition, IL-10 is an important anti-inflammatory cytokine in the body. It is mainly produced by monocytes and can inhibit the synthesis of pro-inflammatory cytokines (such as TNF-α, IL-1β) in macrophages and Th1 T cells (Li et al., 2014). Studies have shown that IL-10 deficient mice can spontaneously form UC, suggesting that IL-10 plays an important role in intestinal immune regulation (Soufli et al., 2016; Wu et al., 2016). Besides, many drugs (e.g. olmesartan and gallic acid) that can alleviate UC have a significant reversal effect on the reduced level of IL-10 in mice with colitis (Saber et al., 2019; Zhu et al., 2019). Therefore, regulating and maintaining the balance between pro-inflammatory and anti-inflammatory cytokines and restoring the body’s immune function are essential for the treatment of UC. Our results showed that the plasma levels of TNF-α and IL-1β were significantly increased and IL-10 was significantly decreased in the model group (**Figure 5**), indicating that the inflammatory response in the model group was significant, which was consistent with the results reported by previous researchers (Saber et al., 2019). As expected, the intervention of HLJDD (9 g/kg and 4.5 g/kg) could significantly reduce the levels of TNF-α and IL-1β in plasma of mice and significantly increase the level of IL-10 (**Figure 5**), indicating that HLJDD has significant anti-inflammatory effect and could suppress abnormal immune response, which was consistent with the report of anti-inflammatory effect of HLJDD (Chen et al., 2016).

Furthermore, in order to investigate the anti-inflammatory mechanism of HLJDD, we examined the NF-κB signaling pathway protein in mouse colon tissue. NF-κB signaling pathway plays an important role in the process of inflammation (Niu et al., 2015). Studies have shown that both in IBD patients and DSS-induced UC mouse models, the NF-κB signaling pathway were abnormally activated (Atreya et al., 2008; Liu et al., 2018). The nuclear factor NF-κB is a heterodimer, and the complex of the two subunits, p50 and p65, is its most common form (Karin and Ben-Neriah, 2000). Under normal conditions, NF-κB is mainly located in the cytoplasm of cells, and it has no transcriptional activity because it forms a complex with inhibitory protein kappa B (IκBα, IκBβ, and IκBε). When the body are stimulated by exogenous substances (such as LPS) or proinflammatory cytokines (e.g., TNF-α, IL-1β), the corresponding receptors on the cell membrane are activated and the signals are transmitted into the cytoplasm, which can phosphorylate IκB kinase (IκK), and then the activated IκK further catalyzes the phosphorylation of IκB. The conformation of IκB is changed after phosphorylation, then it dissociates with the complex of NF-κB and degrades under the induction of ubiquitination, while the free NF-κB translocates into the nucleus. In the nucleus, NF-κB binds with specific DNA sequences and then promotes cell transcription gene, encoding a large number of inflammatory reaction proteins (including TNF-α, IL-1β, and alpha IL-6), thus promoting inflammation (Lopes de Oliveira et al., 2019). Our results showed that compared with NC group, the levels of NF-κB p65, p-IκKα/β, and p-IκBα in colon tissue of model group mice were significantly increased (**Figure 7**), which indicated that the NF-κB signaling pathway in model group mice were significantly activated. HLJDD treatment could significantly reduce the levels of these proteins and has significant anti-inflammatory effect (**Figure 7**), which was consistent with our previous cytokine test results (**Figure 5**). These results indicated that HLJDD could ameliorate DSS-induced inflammatory responses in UC mice by inhibiting NF-κB signaling. In an earlier study that could support our findings, the author demonstrates that HLJDD could inhibit the HMGB-1/TLR4/NF-κB signaling pathway (Xu et al., 2017).

The experimental data showed that besides inflammation, there was significant oxidative stress in colitis mice (Li et al., 2017). Therefore, antioxidant strategies also have great prospects in UC. In this study, we confirmed the presence of significant oxidative stress in DSS-induced UC mice by detecting the oxidative stress parameters of mouse colon tissue. HLJDD intervention could significantly reduce the levels of NO and MDA in colon tissue of UC mice and increase the level of GSH and the activity of SOD antioxidant enzymes (**Figure 6**), suggesting that the therapeutic effect of HLJDD on UC was also derived from its antioxidant effect. Our findings are consistent with earlier studies, suggesting that HLJDD has antioxidant effects (Zhang et al., 2017b).

It is well known that nuclear factor erythroid 2-related factor 2 (Nrf2) signaling pathway is a defense system that mainly regulates the expression of antioxidant proteins in the body. Under normal conditions, Nrf2 is mainly located in the cytoplasm and is combined with Keap1 (a repressor protein of Nrf2) in a ratio of 1:2 (Taguchi et al., 2011). Subsequently, Nrf2 was degraded under the catalysis of Keap1 E3 ubiquitinated linker enzyme and 26S proteasome, which kept the content of Nrf2 in cells at a low level in order to maintain cell homeostasis. Under the stimulation of ROS, RNS, and electrophiles, the degradation of Nrf2 protein was terminated and the binding of Nrf2 to Keap1 was disrupted. Then, the *de novo* synthesized Nrf2 accumulated and translocated into the nucleus. In the nucleus, Nrf-2 heterodimerizes with the small protein Maf or Jun and binds to the antioxidant response element ARE (a conserved gene sequence located in the upstream regulatory region of the gene encoding antioxidant proteins), and then induces the expression of antioxidant proteins (such as HO-1 and SOD) to scavenge oxidants, thereby protecting cells from oxidative stress damage (Harder et al., 2015; Mohan and Gupta, 2018). It is reported that Nrf2 signaling pathway is significantly inhibited in UC rats (Khodir et al., 2019). Consistent with this report, the results of this study showed that the expression of Nrf2 in colon tissues of mice in model group was significantly reduced. Contrary to previous studies, our results also showed that Keap1 levels were significantly reduced in UC mice, and these abnormal changes could be significantly reversed by HLJDD (**Figure 8**). Studies have shown that autophagy is inhibited in colon tissues of IBD patients and experimental animals (Nguyen et al., 2014; Xu et al., 2018; Zheng, 2019). When autophagy was inhibited, p62 (autophagy adapter protein) aggregated and its protein level increased significantly (Bjørkøy et al., 2005). Another study also showed that p62 could bind to Keap1 and promote the degradation of Keap1, thereby enhancing the activity of Nrf2 (Lau et al., 2010; Ichimura et al., 2013). Therefore, we speculate that in order to maintain the intestinal tissue homeostasis under oxidative stress during UC, the degradation of Keap1 is accelerated through p62-dependent pathway in order to compensate for the recovery of antioxidant function. In addition, Zhang et al. partially confirmed our hypothesis; their studies showed that there was significant oxidative stress in the brain of ischemic stroke rats, the expression of p62 was significantly increased, and autophagy was inhibited, and these abnormal changes could be significantly reversed by HLJDD treatment (Zhang et al., 2017b). Therefore, combined with the above antioxidant parameters, we have sufficient evidence that HLJDD could alleviate DSS-induced UC through its antioxidant effect, and its mechanism was partly due to the activation of Nrf2 signaling pathway.

In addition to its important role in the antioxidant process, Nrf2 also plays an active role in the anti-inflammatory process, which has been confirmed in many diseases, including IBD (Sun et al., 2015a; Chen et al., 2017c; Khodir et al., 2019). At present, the interaction between Nrf2 and NF-κB signaling pathway has been confirmed (Wardyn et al., 2015). Keap1 can increase the stability of IκB by promoting the degradation of IκK, which in turn inhibits the activation of NF-κB signaling pathway (Mohan and Gupta, 2018). On the other hand, NF-κB p65 competes with Nrf2 for the binding to CBP (a transcriptional co-activator that helps transcription factors such as NF-κB p65 and Nrf2 to initiate transcription) and enhance transcription of downstream genes of NF-κB (Sun et al., 2009). At present, the literature indicates that there are abnormal disorders of Nrf2 and NF-κB signaling pathways in UC mice (Liu et al., 2018; Saber et al., 2019). Interestingly, studies have shown that drugs (e.g., olmesartan and licochalcone A) that activate the Nrf2 signaling pathway and inhibit the NF-κB signaling pathway could significantly improve experimental colitis (Liu et al., 2018; Saber et al., 2019). Therefore, we speculate that regulating abnormal NF-κB and Nrf2 signaling pathways and maintaining their normal levels may be an effective way to treat UC. Our results also confirm that HLJDD therapy could not only significantly inhibit over-activated NF-κB signaling pathway, but also activate Nrf2 signaling pathway to normal level (**Figures 7**, **8**), which could alleviate DSS-induced UC in mice and could be used as a candidate drug for UC treatment.

Loss of intestinal mucosal barrier is considered to be another important inducement for UC formation (Eom et al., 2018). Mucin secreted by goblet cells and intestinal epithelial cells are important components of intestinal mucosal mechanical barrier (Arike et al., 2017). Tight junction (TJ) protein (e.g., ZO-1 and occludin) is an important parameter reflecting intestinal epithelial cell barrier (Lee, 2015). HLJDD could significantly improve DSS-induced pathological damage (**Figure 4**). Therefore, we further explored the effect of HLJDD on the intestinal mucosal mechanical barrier in mice. WB and immunofluorescence results showed that compared with the model group, HLJDD treatment significantly increased the expression of ZO-1 and occludin in colon tissue of UC mice induced by DSS (**Figures 9B**, **10**). In addition, HLJDD intervention significantly inhibited the decrease of DSS-induced mucin secretion (**Figure 9A**). These results indicate that HLJDD could protect intestinal mucosa by increasing the expression of tight junction protein and secretion of mucin. However, its detailed molecular regulation mechanism needs further study.

Finally, it is worth mentioning that we have established a stable and reliable HPLC detection method for 13 active ingredients in HLJDD, which could be used for quality monitoring in HLJDD production. Furthermore, to explore the specific contribution of 13 active ingredients to inhibiting NF-κB signaling pathway, activating Nrf2 signaling pathway and mucosal protection. We searched the literature related to the pharmacological mechanism of these 13 active ingredients. Encouragingly, the literature shows that 12 active ingredients could exert anti-inflammatory effects by inhibiting the NF-κB signaling pathway (Sun et al., 2015b; Li et al., 2016b; Wang et al., 2016b; Yan et al., 2017; Chen et al., 2017a; Chen et al., 2017b; Chen et al., 2018; Guo et al., 2018; Qiu et al., 2018; Lu et al., 2019; Luan et al., 2019; Pan et al., 2019), while only 8 active components exert antioxidant effects by activating the Nrf2 signaling pathway (Li et al., 2016a; Chen et al., 2018; Fang et al., 2018; Luo et al., 2018; Wang et al., 2018; Chen et al., 2019; Jia et al., 2019; Jiang et al., 2019) (**Figure 11**). Besides, there are six active ingredients that exert mucosal protective effects by restoring tight junction protein expression (Zhu et al., 2012; Bribi et al., 2016; Ruan et al., 2016; Chen et al., 2017a; Peng et al., 2018; Yu et al., 2018) (**Figure 11**). In addition, up to now, nine active ingredients have been proved to significantly alleviate experimental UC (Jiang et al., 2015; Sun et al., 2015b; Wang et al., 2016b; Zhang et al., 2017a; Zhang et al., 2017c; Luo et al., 2018; Gao et al., 2019; Li et al., 2019; Mai et al., 2019) (**Figure 11**). Therefore, combined with the results of this study, we have sufficient evidence that HLJDD could significantly alleviate DSS-induced UC in mice by inhibiting NF-κB signaling pathway, activating Nrf2 signaling pathway, and mucosal protection (**Figure 11**). More interestingly, six active ingredients (geniposide, berberine, baicalin, coptisine, chlorogenic acid, and wogonin) can inhibit NF-κB signaling pathway, activate Nrf2 signaling pathway and protect mucosa, and can be used to alleviate UC (**Figure 11**). Besides, the contents of geniposide, berberine, baicalin, and coptisine were higher in HLJDD (**Figure 2**). Hence, we speculate that these four active ingredients can be used to constitute HLJDD “component prescription” for UC treatment, but the efficacy still needs further experimental verification. In addition, as far as the side effects of HLJDD are concerned, only bacterial reverse mutation test (Ames test) and chromosome aberration tests show that HLJDD has potential genotoxicity, and rats will show some sub-chronic toxicity (e.g., reduced number of red blood cells, increased number of platelets and reticulocyte) after 13 weeks of continuous oral administration of HLJDD (Lee et al., 2014; Jin et al., 2017). Therefore, as a short-term treatment for acute UC, HLJDD seems to be safer than many drugs for UC. Nevertheless, the composition of HLJDD is complex, and the total content of these 13 active ingredients only accounts for 22.77% of HLJDD, indicating that there are still a large number of unknown ingredients in HLJDD. Therefore, we are screening and identifying the anti-ulcer active fraction of HLJDD, which will be reported in next studies.

In conclusion, the results of this study suggest that HLJDD could significantly alleviate the clinical symptoms and pathological damage of UC mice by inhibiting NF-κB signaling pathway, activating Nrf2 signaling pathway, and protecting intestinal mucosa, which could be used as a candidate drug for the treatment of UC.

## Data Availability Statement

The datasets generated for this study are available on request to the corresponding author.

## Ethics Statement

The animal study was reviewed and approved by Animal Ethics Committee of Gansu Agricultural University and the Animal Protection and Utilization Committee.

## Author Contributions

ZY and YW conceived and designed the experiments. ZY analyzed and interpreted the data. ZY wrote the manuscript. ZY and LY performed the experiments. XZ performed tissue sample collection. ZY and LY performed pathological sections. PJ and YH read and revised the manuscript.

## Funding

This research was supported by China Agriculture Research System-37 (CARS-37) and The Fostering Foundation for the Excellent Ph.D. Dissertation of Gansu Agricultural University (YB2017004).

## Conflict of Interest

The authors declare that the research was conducted in the absence of any commercial or financial relationships that could be construed as a potential conflict of interest.

## Abbreviations

HLJDD, Huang-lian-Jie-du Decoction; UC, ulcerative colitis; DSS, dextran sodium sulfate; SASP, sulfasalazine; HPLC, High Performance Liquid Chromatography; DAI, disease activity index; MPO, myeloperoxidase; TNF-α, tumor necrosis factor-alpha; IL, interleukin; GSH, reduced glutathione; NO, nitric oxide; SOD, superoxide dismutase; MDA, malondialdehyde; ROS, reactive oxygen species; RNS, reactive nitrogen species; NF-κB, nuclear transcription factor kappa B; p-IκKα/β, phosphorylated IκB kinase alpha/beta; phosphorylated p-IκBα, inhibitory protein kappa B alpha; Nrf2, nuclear factor erythroid 2-related factor 2; Keap1, Kelch-like ECH-associated protein 1; ZO-1, zona occludens protein 1.
