# Supplementary material for: Huang-Lian-Jie-Du Decoction Ameliorates Acute Ulcerative Colitis in Mice via Regulating NF-κB and Nrf2 Signaling Pathways and Enhancing Intestinal Barrier Function
Source: Front Pharmacol. 2019 Nov 21;10:1354. doi: 10.3389/fphar.2019.01354 (PMC6900672; doi:10.3389/fphar.2019.01354)
Supplement: Supplementary file 1 [file Presentation_1.pdf]

frontiers

Supplementary Material

1 Supplementary Tables

Table S1 Precision test results of peak area and retention time of 13 active ingredients in HLJDD

Type	Relative standard deviation (RSD) values of peak area and retention time of active ingredient (%)												
	Phellodendrine	Chlorogenic acid	Magnoflorine	Geniposide	Coptisine	Epiberberine	Jatrorrhizine	Berberine	Palmatine	Baicalin	Wogonoside	Wogonin	Oroxylin A
Peak area	0.8194	0.9063	0.6703	0.9695	1.2144	1.0967	1.1884	1.2289	1.0306	1.5169	0.9495	1.2810	1.9378
Retention time	0.0212	0.0118	0.0227	0.0195	0.0710	0.0730	0.0456	0.0347	0.0310	0.0161	0.0223	0.0220	0.0219

Table S2 Stability test results of peak area and retention time of 13 active ingredients in HLJDD

Type	Relative standard deviation (RSD) values of peak area and retention time of active ingredient (%)												
	Phellodendrine	Chlorogenic acid	Magnoflorine	Geniposide	Coptisine	Epiberberine	Jatrorrhizine	Berberine	Palmatine	Baicalin	Wogonoside	Wogonin	Oroxylin A
Peak area	1.4867	0.9831	0.8984	0.7714	1.9868	0.6742	1.0637	3.6055	0.9774	4.2808	0.9211	1.3347	2.9781
Retention time	0.0283	0.0293	0.0301	0.0480	0.0863	0.0888	0.0512	0.0364	0.0298	0.0197	0.0263	0.0278	0.0211

Table S3 Repeatability test results of peak area and retention time of 13 active ingredients in HLJDD

Type	Relative standard deviation (RSD) values of peak area and retention time of active ingredient (%)												
	Phellodendrine	Chlorogenic acid	Magnoflorine	Geniposide	Coptisine	Epiberberine	Jatrorrhizine	Berberine	Palmatine	Baicalin	Wogonoside	Wogonin	Oroxylin A
Peak area	4.1781	0.7133	0.6152	1.7563	2.7057	1.5230	0.9577	1.1375	0.8636	2.7862	0.5916	1.6798	3.4687
Retention time	0.0205	0.0512	0.0253	0.0701	0.0630	0.0776	0.0524	0.0362	0.0308	0.0456	0.0490	0.0859	0.0359

Table S4 Result of sample recovery test of 13 active ingredients in HLJDD

Sample NO.	Sample recovery rate (%)												
	Phellodendrine	Chlorogenic acid	Magnoflorine	Geniposide	Coptisine	Epiberberine	Jatrorrhizine	Berberine	Palmatine	Baicalin	Wogonoside	Wogonin	Oroxylin A
Sample 1	90.2184	93.7096	94.9414	97.0334	97.4727	95.1455	97.8487	99.7714	90.3747	93.6284	92.3687	93.4213	87.6979
Sample 2	95.1010	95.9898	99.1166	103.1939	93.9099	95.7816	96.4486	98.5451	89.4230	92.1066	89.5190	85.9954	91.4338
Sample 3	96.5762	94.0082	101.5908	99.1830	102.8677	101.1339	96.1167	100.6407	94.7688	89.9628	98.1792	95.9405	94.5604
Sample 4	102.4939	92.5532	94.9190	98.1954	97.6736	97.0484	98.6925	99.1372	98.0616	96.0550	94.0008	91.5284	90.9385
Sample 5	98.5695	95.1646	97.9018	97.5266	98.8344	107.8180	97.6055	98.7856	99.7452	91.7818	102.5074	88.9478	89.8301
Mean	96.5918	94.2851	97.6939	99.0264	98.1516	99.3855	97.3424	99.3760	94.4746	92.7069	95.3150	91.1667	90.8921
SD	4.0395	1.1905	2.5507	2.2047	2.8771	4.7030	0.9434	0.7552	4.0758	2.0399	4.5593	3.4545	2.2398
RSD	4.1821	1.2627	2.6109	2.2264	2.9313	4.7321	0.9691	0.7599	4.3142	2.2003	4.7834	3.7892	2.4642

Table S5 Content of 13 active ingredients in HLJDD

Sample NO.	Content (mg/g)												
	Phellodendrine	Chlorogenic acid	Magnoflorine	Geniposide	Coptisine	Epiberberine	Jatrorrhizine	Berberine	Palmatine	Baicalin	Wogonoside	Wogonin	Oroxylin A
Sample 1	3.9141699	1.0995556	6.7512745	72.93132	14.096699	8.915915	9.4558497	53.201869	19.674908	18.599216	17.73568	2.4908105	0.2711327
Sample 2	3.8387778	1.0825294	6.7836732	72.844471	14.165706	8.969098	9.4385033	53.445092	19.801863	18.636784	17.515451	1.9913595	0.2422078
Sample 3	3.7298627	1.0579281	6.7117386	71.371882	13.913124	8.8318889	9.3140523	52.598752	19.569817	18.493614	17.357902	2.0246471	0.2721484
Mean	3.8276035	1.0800044	6.7488954	72.382558	14.05851	8.905634	9.4028017	53.081904	19.682196	18.576538	17.536344	2.168939	0.2618296
SD	0.0926603	0.0209283	0.0360263	0.8763471	0.1305496	0.0691799	0.0773471	0.4357368	0.1161944	0.0742301	0.1897535	0.2792453	0.0170006

Table S6 The consumption volume of 3.5% DSS drinking water in mice of each group

Day	NC (ml)	Model (ml)	SASP (ml)	HLJDD-H (ml)	HLJDD-M (ml)	HLJDD-L (ml)
1	37.5	38.0	39.5	34.5	36.0	39.0
2	39.0	41.0	40.0	38.5	38.0	37.0
3	36.0	42.5	39.0	41.0	38.5	33.5
4	37.5	38.5	41.5	40.5	41.0	40.0
5	35.0	37.0	33.5	35.0	33.0	36.0
6	35.5	32.5	29.5	27.5	31.5	29.5
7	36.0	24.5	27.5	30.0	28.0	32.0
Mean	36.64	36.29	35.79	35.29	35.14	35.29
SD	1.41	6.09	5.60	5.15	4.53	3.81

Table S7 The consumption of feed in mice of each group

Day	NC (g)	Model (g)	SASP (g)	HLJDD-H (g)	HLJDD-M (g)	HLJDD-L (g)
1	24.2	22.9	23.3	22.6	23.5	22.3
2	23.1	21.1	22.8	24.2	23.7	23.7
3	24.0	20.6	21.2	21.6	22.5	27.6
4	24.2	20.2	22.9	21.4	25.2	24.9
5	22.7	19.9	21.4	20.6	23.7	20.9
6	21.6	17.2	18.6	19.2	20.7	19.3
7	23.2	12.1	14.9	18.9	17.7	15.1
Mean	23.29	19.14	20.73	21.21	22.43	21.97
SD	0.95	3.54	3.02	1.86	2.50	4.06

2 Supplementary Figures

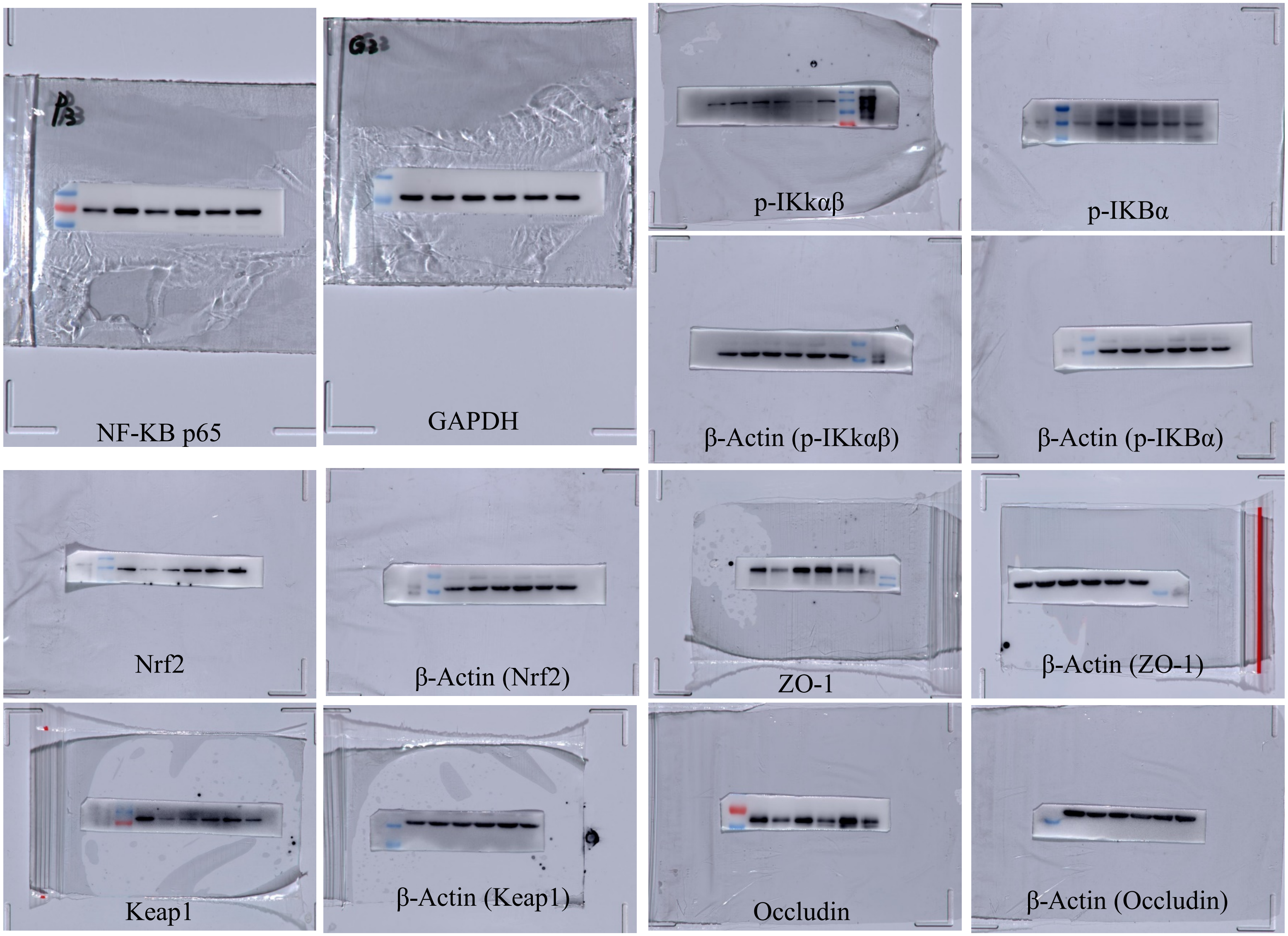

Supplementary Figure S1. Raw figures of WB

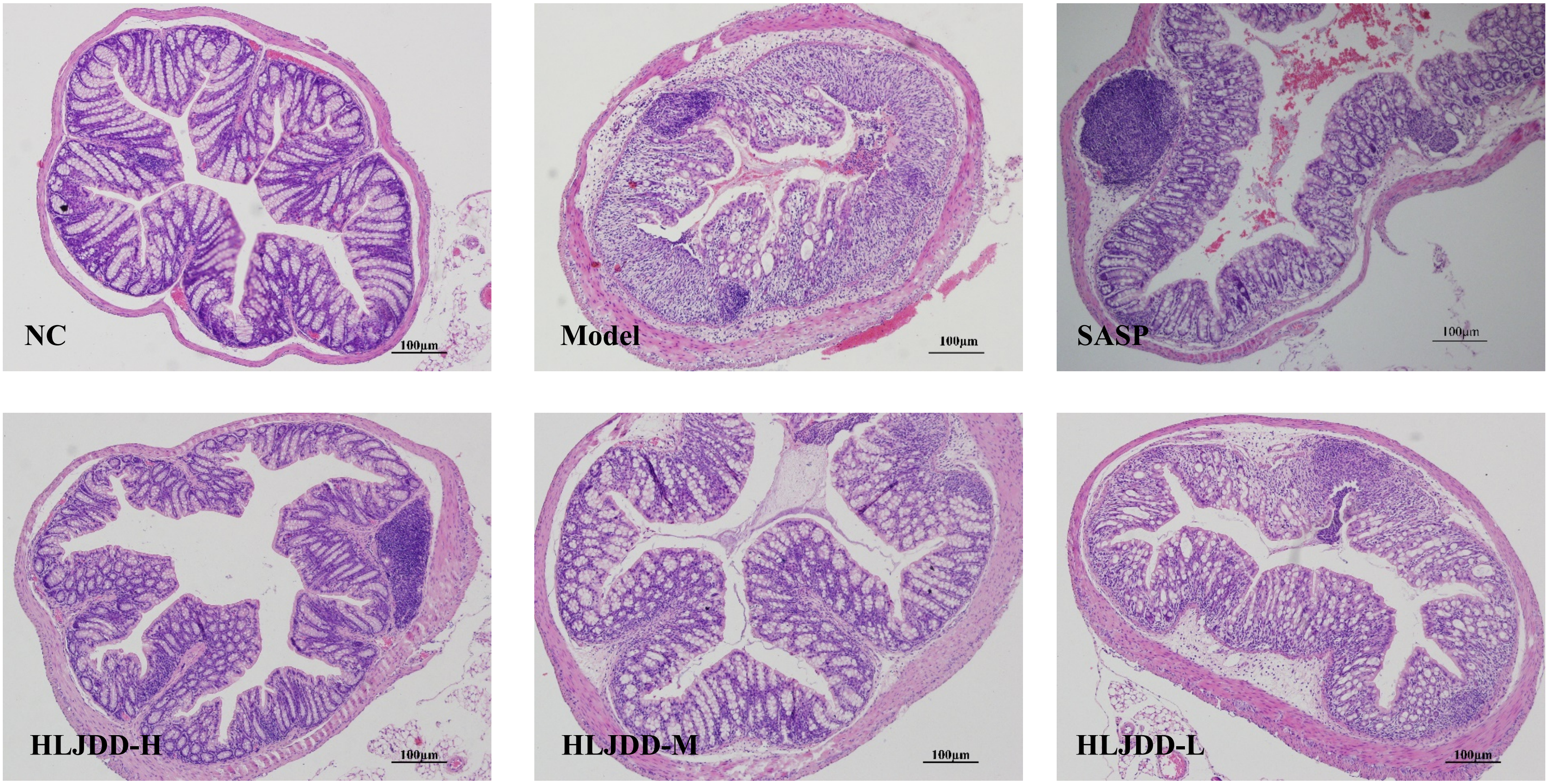

Supplementary Figure S2. Raw figures of HE staining (100 × magnification)

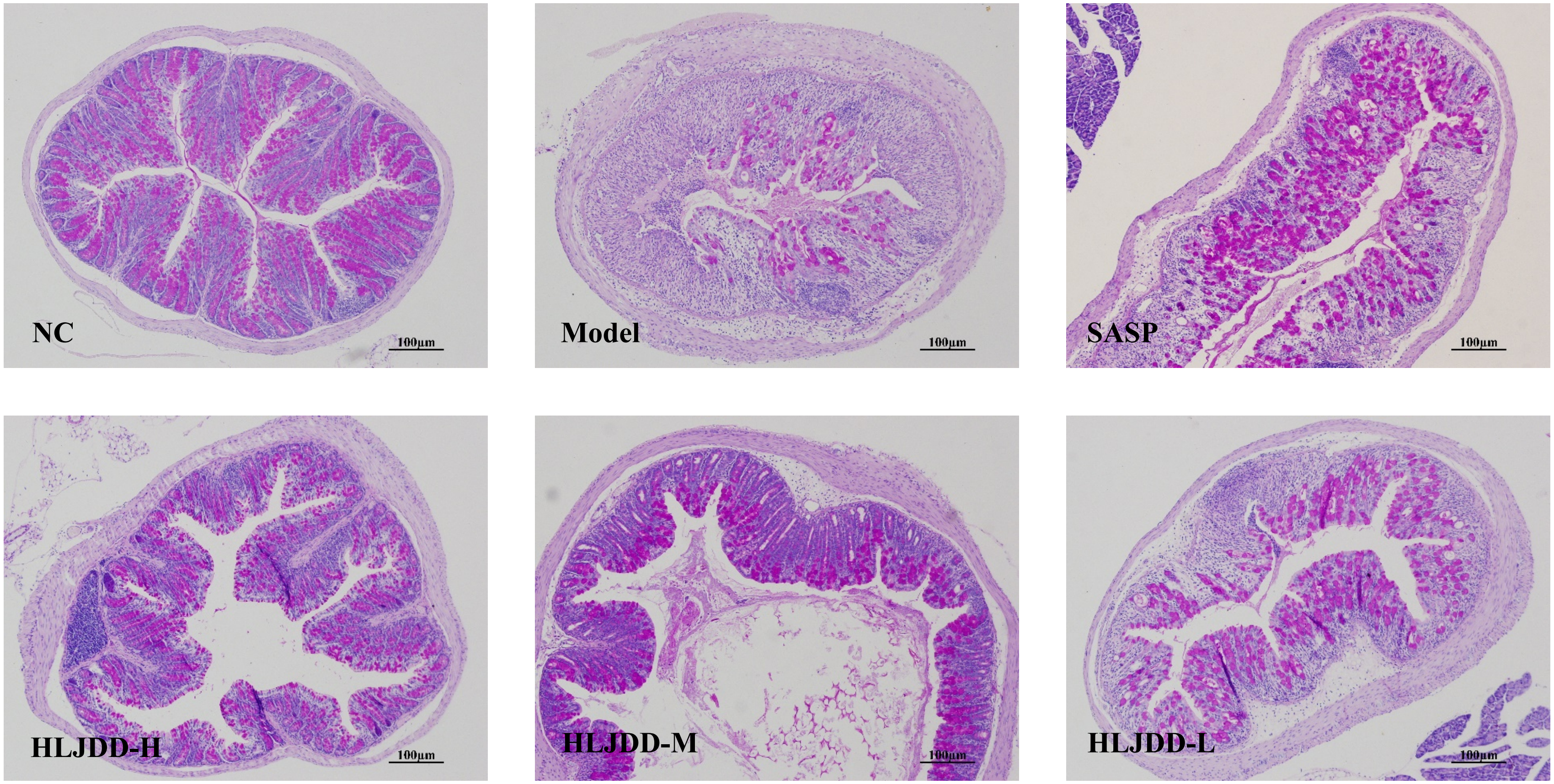

Supplementary Figure S3. Raw figures of PAS staining (100 × magnification)
